# Recent Advances in Metabolically Engineered Microorganisms for the Production of Aromatic Chemicals Derived From Aromatic Amino Acids

**DOI:** 10.3389/fbioe.2020.00407

**Published:** 2020-05-05

**Authors:** Yu-Ping Shen, Fu-Xing Niu, Zhi-Bo Yan, Lai San Fong, Yuan-Bin Huang, Jian-Zhong Liu

**Affiliations:** Guangdong Province Key Laboratory of Improved Variety Reproduction in Aquatic Economic Animals, Biomedical Center, School of Life Sciences, Institute of Synthetic Biology, Sun Yat-sen University, Guangzhou, China

**Keywords:** aromatic amino acid derivatives, stilbenes, benzylisoquinoline alkaloids, metabolic engineering, microorganism, strain construction

## Abstract

Aromatic compounds derived from aromatic amino acids are an important class of diverse chemicals with a wide range of industrial and commercial applications. They are currently produced via petrochemical processes, which are not sustainable and eco-friendly. In the past decades, significant progress has been made in the construction of microbial cell factories capable of effectively converting renewable carbon sources into value-added aromatics. Here, we systematically and comprehensively review the recent advancements in metabolic engineering and synthetic biology in the microbial production of aromatic amino acid derivatives, stilbenes, and benzylisoquinoline alkaloids. The future outlook concerning the engineering of microbial cell factories for the production of aromatic compounds is also discussed.

## Introduction

Aromatic compounds are an important class of diverse chemicals. They have extensive applications in the production of various solvents, plastics, fine chemicals, food and feed additives, nutraceuticals, and pharmaceuticals (Averesch and Kromer, 2018; Wang J. et al., 2018). Moreover, many aromatic products have various biological activities and are widely used in pharmaceutical industries. Typically, aromatic compounds are chemically synthesized from petroleum-derived feedstocks, such as benzene, xylene, and toluene. With the progressive exhaustion of traditional feedstocks and the continuous deterioration of the environment, the biotechnological production of aromatic chemicals from renewable sugar feedstock in an eco-friendly manner has received considerable attention as a promising alternative for the synthesis of aromatic compounds.

**Figure 1:**
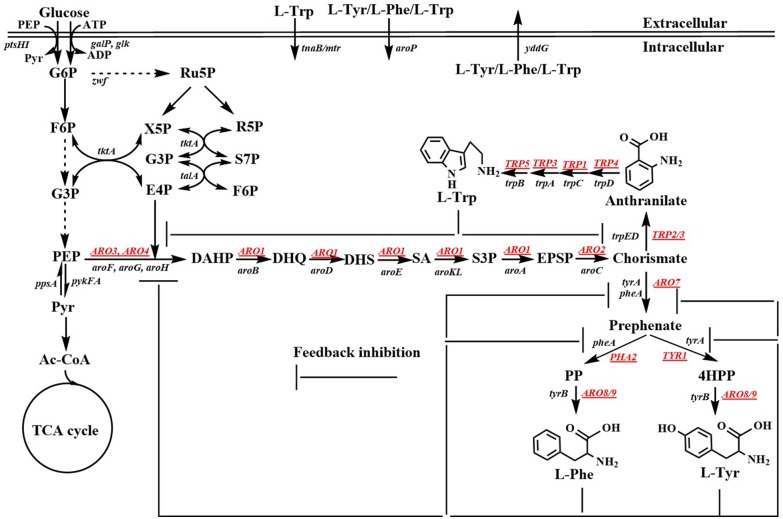
Biosynthetic pathway of aromatic amino acids. Black, *Escherichia coli* genes; Underlined genes, *Saccharomyces cerevisiae* genes; G6P, glucose-6-phosphate; F6P, fructose-6-phosphate; G3P, glyceraldehde-3-phosphate; PEP, phosphoenolpyruvate; Pyr, pyruvate; Ac-CoA, acetyl-CoA; TCA cycle, tricarboxylic acid cycle; Ru5P, ribulose-5-phosphate; X5P, xylulose-5-phosphate; R5P, ribose-5-phosphate; S7P, sedoheptulose-7-phosphate; E4P, D-erythrose 4-phosphate; DAHP, 3-deoxy-D-arabino-heptulosonate-7-phosphate; DHQ, 3-dehydroquinate; DHS, 3-dehydroshikimate; SA; shikimic acid; S3P, shikimate-3-phosphate; EPSP, 5-enolpyruvylshikimate 3-phosphate; PP, phenylpyruvate; 4HPP, 4-hydroxyphenylpyruvate; L-Phe, L-phenylanine; L-Tyr, L-tyrosine; L-Trp, L-tryptophan. *ptsHI*, phosphocarrier protein HPr and PTS enzyme I genes; *galP*, galactose, H (+) symporter gene; *glk*, glucokinase gene; *zwf*, glucose-6-phosphate dehydrogenase gene; *tkt*, transketolase 1 gene; *tal*, transaldolase A gene; *ppsA*, phosphoenolpyruvate synthase; *pykFA*, pyruvate kinase I and II genes; *aroF/aroG/aroH*, 3-deoxy-D-arabino-heptulosonate-7-phosphate synthase genes; *aroB*, 3-dehydroquinate synthase gene; *aroD*, 3-dehydroquinate dehydratase gene; *aroE*, dehydroshikimate reductase gene; *aroK/aroL*, shikimate kinase genes; *aroA*, EPSP synthase gene; *aroC*, chorismate synthase gene; *pheA/tyrA*, chorismate mutase/prephenate dehydrogenase gene; *tyrB*, aromatic-amino-acid transaminase gene; *trpD/trpE*, anthranilate synthase gene; *trpC*, phosphoribosylanthranilate isomerase gene; *trpA*, indoleglycerol phosphate aldolase gene; *trpB*, tryptophan synthase gene; *tnaB/mtr*, tryptophan: H (+) symporter gene; *aroP*, aromatic amino acid: H (+) symporter gene; *yddG*, aromatic amino acid exporter gene; *ARO3/ARO4*, 3-deoxy-D-arabino-heptulosonate-7-phosphate synthase genes; *ARO2*, chorismate synthase; *ARO7*, chorismate mutase gene; *TYR1*, prephenate dehydrogenase gene; *ARO8/ARO9*, aromatic acid aminotransferase gene; *PHA2*, prephenate dehydratase gene; TRP2/TRP3, anthranilate synthase gene; *TRP4*, anthranilate phosphoribosyl transferase gene; *TRP1*, N-(5'-phosphoribosyl)-anthranilate isomerase gene; *TRP3*, indole-3-glycerol phosphate synthase gene; *TRP5*, tryptophan synthetase gene.

Aromatic compounds can be produced by various plants, algae, fungi, and bacteria via the shikimate pathway. The shikimate pathway begins with the condensation of phosphoenolpyruvate (PEP) in the Embden-Meyerhof-Parnas pathway (EMP) and that of D-erythrose 4-phosphate (E4P) in the pentose phosphate pathway (PPP) to form 3-deoxy-D-arabino-heptulosonate-7-phosphate (DAHP). Six additional enzymatic steps are necessary for the synthesis of chorismite, which is converted to phenylpyruvate (PP) or 4-hydroxyphenylpyruvate (4HPP) via prephenate, through the Phe-Tyr branch that is catalyzed by chorismate mutase/prephenate dehydratase (PheA) or the chorismate mutase/prephenate dehydrogenase (TyrA) complex. Chorismate is also converted to anthranilate (AA) by the Trp branch in a reaction catalyzed by the anthranilate synthase (TrpED) complex. Finally, Phe, Tyr, and Trp are synthesized via their own biosynthetic pathways using PP, 4HPP, or anthranilate, respectively, as precursors (Figure 1). The enzymes and regulations involved in the aromatic amino acid biosynthetic pathway have been well-studied and characterized. Cao et al. (2020) well-summarized the transcriptional regulation mechanism. Herein, we only outline the feedback inhibition mechanism in *Escherichia coli* and *Saccharomyces cerevisiae* (Figure 1). In *E. coli*, DAHP synthase, which is encoded by *aroF, aroG*, and *aroH* is feedback-inhibited by L-tyrosine (L-Tyr), L-phenylalanine (L-Phe), and L-tryptophan (L-Trp), respectively. Additionally, *aroF, aroG*, and *aroH* are negatively regulated by TyrR, a transcriptional regulatory protein. Chorismate mutase or prephenate dehydrogenase encoded by *tyrA* or *pheA* is also regulated through feedback inhibition by L-Tyr or L-Phe, respectively. DAHP synthase, 3-dehydroquinate (DHQ) synthase (AroB), shikimate kinase (AroKL), 5-enolpyruvoylshikimate 3-phosphate (EPSP) synthase (AroA), and choirmate synthase (AroC) are the rate-limiting enzymes in the shikimate pathway (Dell and Frost, 1993). In *S. cerevisiae*, DAHP synthase is encoded by *ARO4* and *ARO3*, and regulated through feedback inhibition by L-Tyr and L-Phe. ARO1 is a pentafunctional enzyme that catalyzes steps 2 through 6 during the biosynthesis of chorismate.

With the developments of metabolic engineering and synthetic biology, significant advances have been made by engineering the shikimate pathway to produce natural or non-natural aromatic compounds at the gram level in microorganisms, especially in *E. coli* and *S. cerevisiae* (Averesch and Kromer, 2018; Wang J. et al., 2018; Wu et al., 2018; Huccetogullari et al., 2019). Here, we provide a comprehensive and systematic overview of the recent advancements in metabolic engineering and synthetic biology used to achieve the microbial production of aromatic compounds derived from aromatic amino acids. These aromatic compounds include aromatic amino acid derivatives, stilbenes, and benzylisoquinoline alkaloids. More aromatic amino acid derivatives were reviewed in this study. Some groups gave some good reviews about the metabolic engineering strategies and the progress for the production of aromatic amino acid derivatives before 2019 (Averesch and Kromer, 2018; Huccetogullari et al., 2019; Cao et al., 2020). Overexpression of the feedback-resistant mutants and transketolase are the common strategies for engineering microorganisms to produce aromatic ammonic acids. These strategies will not be specially mentioned. To avoid repeating, we will focus on the production of aromatic compounds using metabolic engineering microorganisms.

## Production of Aromatic Amino Acid Derivatives

Aromatic amino acid derivatives are a class of important aromatic compounds derived from aromatic amino acids.

### Phenylalanine Derivatives

**Figure 2:**
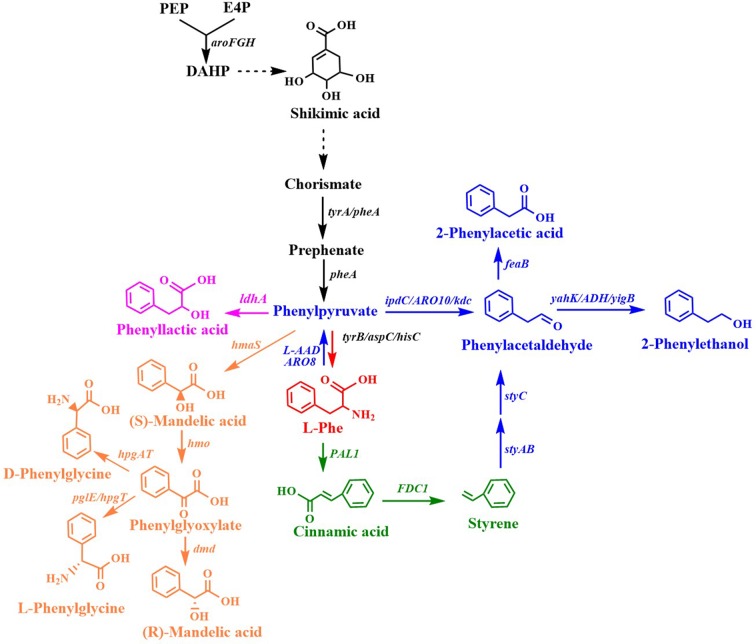
Biosynthesis of L-phenylalanine derivatives. *ldhA*, lactate dehydrogenase gene; *ipdC/ARO10/kdc*, phenylpyruvate decarboxylase gene; *yahK/ADH6/yigB/adh*, aldehyde reductase; *feaB*, phenylacetaldehyde dehydrogenase gene; *L-AAD*, L-amino acid deaminase gene; *ARO8*: *PAL1*, phenylalanine ammonia lyase gene; *FDC1*, ferulic acid decarboxylase 1 gene; *styAB*, styrene monooxygenase genes; *styC*, styrene oxide isomerase gene; *hmaS*, S-mandelate synthesis gene; *hmo*, l-4-hydroxymandelate oxidase gene; *hpgT*, l-4-hydroxyphenylglycine transaminase gene; *pglE*, phenylglycine aminotransferase; *hpgAT*, D-(4-Hydroxy)phenylglycine aminotransferase gene; *dmd, D-mandelate dehydrogenase gene*.

Many aromatic chemicals can be derived from L-Phe or its precursor phenylpyruvate by a suitable biosynthetic pathway (Figure 2, Table 1). Phenylpyruvate derivatives, such as phenyllactic acid (PLA), 2-phenylethanol (2PE), 2-phenylacetic acid and mandelic acid, are synthesized using phenylpyruvate as a common precursor (Figure 2, Table 1). **PLA** is an antifungal compound. The replacement of the *acs* and *mtlA* with the *Cupriavidus necator* JCM20644 lactate dehydrogenase gene *ldhA* under the control of the T7 promoter in the L-Phe producing *E. coli* strain resulted in the production of 6.4 mM of L-PLA from glucose in shake flask cultures (Koma et al., 2012). Overexpression of *Wickerhamia fluorescens* phenylpyruvate reductase gene (*ppr*), native feedback-resistant *aroG*^*fbr*^ and *pheA*^*fbr*^ in the L-Phe producing strain *E. coli* NST37 resulted in the production of 29.2 g/L of D-PLA from glucose in 0.4 L bioreactor fed-batch fermentations (Fujita et al., 2013). They also found that the replacement of *W. fluorescens ppr* with lactate dehydrogenase gene (*L-ldh*) from *Pediococcus acidilactici* generated L-PLA. Recently, whole-cell biocatalysis has been successfully used to produce L-PLA from L-Phe or phenylpyruvate. An *E. coli* expressing *Lactobacillus plantarum* lactate dehydrogenase gene (*L-ldh*)*, Proteus mirabilis* L-amino acid deaminase gene (*L-aad)*, and *Candida boidinii* formate dehydrogenase gene (*fdh*) was constructed for the production of L-PLA by using whole-cell biocatalysis (Hou et al., 2019). The resulting strain produced 54.0 g/L of L-PLA from L-Phe with the aid of glucose, which was used as a co-substrate for cofactor regeneration.

**Table 1:** *De novo* production of phenylalanine derivatives by engineered microorganisms.

**Products**	**Host**	**Applications**	**Metabolic engineering strategies**	**Culture conditions**	**Titer**	**Yield**	**References**
L-Phenyllactic acid	*Escherichia coli*	Antifungal compound	Δ*tyrR*::P_T7_-*aroF^*fbr*^-pheA^*fbr*^* Δ*acs*::P_T7_-*ldhA, mtlA*::P_T7_-*ldhA*	Shake-flask fermentation	6.4 mM		Koma et al., 2012
D- Phenyllactic acid	*E. coli* NST37		Expressing *Wickerhamia fluorescens ppr* on a plasmid (pHSG298), and *pheA^*fbr*^-aroG^*fbr*^* on another plasmid	0.4 L bioreactor fed-batch fermentation	29.2 g/L		Fujita et al., 2013
2-Phenylethnol	*E. coli*	Flavor and fragrance	Δ*tyrR::*P_T7_*-aroF^*fbr*^-pheA^*fbr*^* Δ*mtlA::*P_T7_*-ipdC, Δacs::*P_T7_*-yahK ΔfeaB*	Shake-flask fermentation	7.7 mM (940.6 mg/L)		Koma et al., 2012
	*E. coli*		Expressing the P_lac_ promoter-controlled *pheA^*fbr*^-aroF* on a pCL 1920 plasmid. Expressing *adh1-kdc* on a pBR322 origin plasmid	Shake-flask fermentation	285 mg/L		Kang et al., 2014
	*E. coli* ATCC 31884 (NST74) (L-Phe over-producer)		*ΔfeaB Δcrr ΔpykFA*. Expressing *A. thaliana PAL2* and *S. cerevisiae FDC1* on a p15A origin plasmid. Expressing *P. putida styABC* on a ColA origin plasmid	Shake-flask fermentation	1.94 g/L		Machas et al., 2017
	*E. coli* BL21 (DE)		Expressing L-amino acid deaminase and α-keto acid decarboxylase from *Proteus mirabilis* JN458 on a pACYCDuet-1 plasmid. Expressing alcohol dehydrogenase from *P. mirabilis* JN458 and glucose dehydrogenase from *Bacillus subtilis* ATCC 13952 on a pACYCDuet-1 plasmid	Shake-flask bioconversion	2.88 g/L from Phe	97.38% mol/mol	Liu J. B. et al., 2018
	*E. coli* BL21 (DE)		Expressing the T7 promoter-controlled *aroG^*fbr*^* and *pheA^*fbr*^* on a pBBRMCS1 plasmid. Expressing the T7 promoter-controlled *S. cerevisiae kdc, E. coli yigB* and *S. cerevisiae ARO8* on a pET28a plasmid	Shake-flask fermentation	1016 mg/L		Guo et al., 2018
	*Kluyveromyces marxianus*		An evolved *K. marxianus* strain resistant to the phenylalanine analog, *p*-fluorophenylalanine. Plasmid-expressing *S. cerevisiae ARO10* and *ADH2*. Plasmid-expressing *K. marxianus aroG^*fbr*^*	Shake-flask fermentation	1.3 g/L		Kim et al., 2014
	*Saccharomyces cerevisiae*		*ΔALD3*. Expressing *ARO80* on a pRS424ADH plasmid. Expressing *ARO9* on a pRS425ADH plasmid and *ARO10* on a pRS426ADH plasmid	Two-phase Shake-flask fermentation	6.1 g/L	82.5% mol/mol	Kim B. et al., 2014
2-Phenylacetic acid	*E. coli*	Precursor for the chemical synthesis of penicillin G, the atenolol drug, agrochemicals	Δ*tyrR*::P_T7_-aroF^fbr^-pheA^fbr^, *ΔmtlA*::P_T7_-*ipdC, Δacs*::P_T7_-*feaB, Δ*tyrA	Shake-flask fermentation	8.8 mM (1198.1 mg/L)		Koma et al., 2012
S-Mandelic acid	*E. coli*	Precursor for the synthesis of penicillins, cephalosporins, anti-obesity agents, and pharmaceuticals with anti-HIV or anti-leukemic activities	Δ*tyrB ΔaspC ΔtyrA ΔtrpE*. Expressing the *tac* promoter-controlled *pheA^*fbr*^* and S-mandelate synthesis gene *hmaS* from *Amycolatopsis orientalis*, and *aroF* promoter-controlled *aroF^*fbr*^* on a plasmid	Shake-flask fermentation	1.02 g/L		Sun et al., 2011
	*E. coli-E. coli*		(S)-Mandelic acid producer L37: Expressing *Aspergillus niger pad1, A. thaliana PAL2*, styrene monooxygenase gene (*SMO*) from *Pseudomonas*sp. VLB120, epoxide hydrolase gene (*SpEH*) from *Sphingomonas* sp. HXN-200, alcohol dehydrogenase gene (*AlkJ*) from *P. putida* GPo1, phenylacetaldehyde dehydrogenase gene (*aldh*) from *E. coli*. L-Phe producing strain: NST74	Coculture whole-well biocatalysis	10 g/L from glycerol		Lukito et al., 2019
R-Mandelic acid	*E. coli*		Δ*tyrB ΔaspC ΔtyrA ΔtrpE*. Expressing tac promoter-controlled *pheA^*fbr*^* and S-mandelate synthesis gene *hmaS* from *A. orientalis*, trc promoter-controlled 4-hydroxymandelate oxidase gene *hmo* from *Streptomyces coelicolor* and D-mandelate dehydrogenase gene *dmd* from *Rhodotorula graminis*, and *aroF* promoter-controlled *aroF^*fbr*^* on a p15A origin plasmid (pSU2718)	Shake-flask fermentation	0.88 g/L		Sun et al., 2011
Mandelic acid	*S. cerevisiae*		*TRP1, aro4*Δ*:: HXT7pr-ARO1, ura3*Δ*::TPI1pr-ARO4^*fbr*^_HXT7pr-coptARO3^*fbr*^, aro10Δ aro8Δ pdc5Δ trp2Δ tyr1Δaro9Δ*	Shake-flask fermentation	236 mg/L	1.7% g/g	Reifenrath and Boles, 2018
*trans-*Cinnamic acid	*E. coli* W3110	Flavoring agent and anti-bacterial compound as well as a precursor of chemicals	*Δcrr ΔtyrR ΔtrpE ΔtyrA ΔpykA*. Expressing *Streptomyces maritimus pal* on pTrc99A plasmid. Expressing the P_tac_-controlled *aroG8/15-ydiB-aroK-pheA^*fbr*^*, the P_pc113_ promoter-controlled *glk* and the P_pc113_ promoter-controlled *galP* on a p15A origin plasmid	Fed-batch fermentation	6.9 g/L		Bang et al., 2018
	*S. cerevisiae* CEN.PK113-7D		*aro10*Δ*::loxP, aro7*Δ*::ARO4^*K*229*L*^, aro4*Δ*::ARO7 ^*G*141*S*^*. Expressing the codon-optimized *pal* from *Photorhabdus luminescens* on high copy plasmid	Shake-flask fermentation	37.9 mg/L		Gottardi et al., 2017
Styrene	*E. coli* ATCC 31884 (NST 74), L-Phe overproducing strain	Synthesizing polymers	Expression *PAL2* from *A. thaliana* on a pACYC184 origin plasmid. Expressing *FDC1* from *S. cerevisiae* on a pBR322 origin plasmid	Shake-flask fermentation	260 mg/L		McKenna and Nielsen, 2011
	*E. coli* ATCC 31884 (NST 74)		Expression *pal1* from *A. thaliana* on a pACYC184 origin plasmid. Expressing *FDC1* from *S. cerevisiae* on a pBR322 origin plasmid	Solvent extraction shake-flask fermentation	836 mg/L		McKenna et al., 2015
	*E. coli* BL (DE3)		Expression *pal1* from *A. thaliana, FDC1* from *S. cerevisiae*, native *ppsA* and *tktA* on a pBR322 origin plasmid. Expressing native *aroF* and *pheA* on a p15A origin plasmid	Solvent extraction shake-flask fermentation	350 mg/L		Liu C. Q. et al., 2018
	*E. coli* 3110		*Δcrr, ΔtyrR, ΔtrpE, ΔtyrA*, and *ΔpykA* Expressing *S. maritimus pal* on a plasmid (pTrc99A, *Ptac–aroG8/15–ydiB–aroK–pheAfbr, dm -P_*pc*113_-glk-T_*lpp*_-P_*pc*113_-galP-T_*lpp*_-P_*BBa*_*J*23100_-ScFDC* on a p15A origin plasmid)	5 L fed-batch fermentation using *in situ* extraction and gas stripping with n-dodecane	5.3 g/L		Lee et al., 2019
	*E. coli* NST74		Chromosomal expressing *PAL2* from *A. thaliana* and *FDC1* from *S. cerevisiae*. iCREATE strategy for 54 transcription regulator	1-L bioreactor fed-batch fermentation using gas stripping strategy with n-dodecane	3.15 g/L		Liang et al., 2020
L-Phenylglycine	*E. coli*	Synthesizing β-lactam antibiotics and taxol	Δ*tyrB, ΔaspC* Expressing *S. coelicolor hmaS, hmo* and *hpgT, aroG^*fbr*^, pheA^*fbr*^, aroK, ydiB*	Shake-flask fermentation	51.6 mg/g DCW		Liu et al., 2014
D- Phenylglycine	*E. coli*		ΔpheA, ΔtyrA, aroF^fbr^, ΔtyrR, Δ*tyrB, ΔaspC* Expressing *A. orientalis hmaS, S. coelicolor hmo, Pseudomonas putida hpgT* and *pheA^*fbr*^* on a plasmid	Shake-flask fermentation	102 mg/g DCW		Muller et al., 2006

**2PE**, which has high-value as flavor and fragrance compound, has a wide range of applications in the cosmetic, perfumery, and food industries. The indol-3-pyruvate/phenylpyruvate decarboxylase gene *ipdC* from *Azospirillum brasilense* NBRC102289 was introduced into a Phe overproducing *E. coli* strain, resulting in the production of 2PE (Figure 2) (Koma et al., 2012). The chromosomal overexpression of *A. brasilense ipdC* and *E. coli yahK*, and the deletion of *feaB* resulted in the production of 7.7 mM (940.6 mg/L) of 2PE from glucose in shake flask cultures (Koma et al., 2012). The overexpression of the phenylpyruvate decarboxylase gene *kdc* from *Pichia pastoris* GS115 and alcohol dehydrogenase (ADH) gene *adh1* from *S. cerevisiae* S288c in *E. coli* resulted in the production of 2PE (Kang et al., 2014). The co-overexpression of the four genes *kdc, adh1, pheA*^*fbr*^, and *aroF* improved 2PE production to 285 mg/L from glucose in shake flask cultures. A novel route (styrene-derived pathway) has been established for 2PE production in *E. coli* (Machas et al., 2017). The styrene-derived pathway comprised *PAL2* from *Arabidopsis thaliana, FDC1* from *S. cerevisiae*, and *styABC* from *Pseudomonas putida* S12. The introduction of the styrene-derived pathway, as well as the deletion of the competing pathways (*feaB, crr*, and *pfkFA*) resulted in the production of 1.94 g/L of 2PE from glucose in shake flask cultures. The results also demonstrated that the styrene-derived pathway more efficiently produced 2PE than the Ehrlich pathway. The pathway for 2PE production, which involves L-amino acid deaminase, α-keto acid decarboxylase, and ADH, was mimicked from the 2PE natural producer *P. mirabilis* JN458 (Liu J. B. et al., 2018). The recombinant *E. coli* harboring this *P. mirabilis* pathway produced 2.88 g/L 2PE from Phe with a molar yield of 97.38%. The plasmid-based overexpression of *S. cerevisiae kdc, E. coli yigB*, and *S. cerevisiae aro8*, as well as the enhancement of the expression of the feedback-resistant *aroG*^*fbr*^ and *pheA*^*fbr*^ in *E. coli* resulted in the production of 1,016 mg/L 2PE from glucose (Guo et al., 2018). After the introduction of alcohol acetyltransferase gene *ATF1* from *S. cerevisiae* into the 2PE producer, 2-phenylethyl acetate was produced and achieved 687 mg/L. *Kiuyveromyces marxianus, S. cerevisiae*, and *P. pastoris* are typical yeast systems that have been engineered to synthesize 2PE. The phenylpyruvate decarboxylase (ARO10) and ADH (ADH2) genes of *S. cerevisiae* were expressed in an evolved *K. marxianus* that was resistant to the phenylalanine analog, *p*-fluorophenylalanine, which resulted in 2PE production (Kim et al., 2014). The overexpression of the feedback-resistant *aroG*^*fbr*^ from *K. marxianus* further increased the production of 2PE from glucose to 1.3 g/L. In *S. cerevisiae*, the overexpression of *ARO9, ARO10*, and *ARO80* (the latter is a transcription activator of *ARO9* and *ARO10*), and the deletion of *ALD3* improved 2PE production (Kim B. et al., 2014). After optimizing the fermentation conditions, the engineered *S. cerevisiae* produced 6.1 g/L of 2PE from glucose with a molar yield of 82.5%. *ARO8*, which encodes aromatic aminotransferase I, was identified to be a transcriptional regulator of *ARO10* (Romagnoli et al., 2015). The deletion of *ARO8* improved 2PE production in *S. cerevisiae* (Romagnoli et al., 2015). *P. pastoris*, a type of the methylotrophic yeast, can rapidly grow to extremely high cell densities in very simple and defined media. *P. pastoris* can naturally synthesize low levels of 2PE. Overexpression of the biosynthetic pathway of 2PE from *S. cerevisiae* (*ARO10-ADH6*) in *P. pastoris* enhanced the 2PE titer to 416 mg/L from 26 mg/L (Kong et al., 2020). Increasing the availability of the precursor of phenylpyruvate furtherly improved the 2PE titer to 1,169 mg/L by overexpressing *S. cerevisiae ARO8, E. coli aroG*^*fbr*^ and *pheA*^*fbr*^.

**2-Phenylacetic acid** and 4-hydroxyphenylacetic acid (4HPAA) are intermediates in the chemical synthesis of penicillin G, atenolol, agrochemicals, and other compounds. The production of 2-phenylacetic acid was achieved by the overexpression of endogenous *feaB* and *ipdC* from *A. brasilense* NBRC102289 in *E. coli* using the Ehrlich pathway intermediates phenylacetaldehyde as precursors (Figure 2) (Koma et al., 2012). The overexpression of *A. brasilense ipdC* and *E. coli feaB*, as well as the deletion of *tyrA* in the Phe overproducing *E. coli* strain resulted in the *de novo* production of 8.8 mM (1,198.1 mg/L) of 2-phenylacetic acid in shake flask cultures (Koma et al., 2012).

Mandelic acid and 4-hydroxymandelic acid (4HMA) are valuable aromatic fine chemicals used as precursors for the production of pharmaceuticals, flavors, and cosmetics. **Mandelic acid** has been widely used in the synthesis of semisynthetic penicillins, cephalosporins, anti-obesity agents, and pharmaceuticals with anti-HIV or anti-leukemic activities, as well as for the resolution of racemic alcohols and amines (Reifenrath and Boles, 2018). The biosynthetic pathway for mandelic acid production has been successfully established in *E. coli* (Sun et al., 2011) and *S. cerevisiae* (Reifenrath and Boles, 2018). The biosynthetic pathway for mandelic acid (Figure 2) was first established in *E. coli* by introducing the S-mandelate synthesis gene *hmaS* from *Amycolatopsis orientalis* (Sun et al., 2011). The overexpression of *hmaS* from *A. orientalis* and the feedback-resistant *pheA*^*fbr*^ and *aroF*^*fbr*^, along with the deletion of the competing pathways resulted in the *de novo* production of 1.02 g/L of S-mandelic acid in shake flask cultures. The co-expression of the 4-hydroxymandelate oxidase gene *hmo* from *Streptomyces coelicolor* and D-mandelate dehydrogenase gene *dmd* from *Rhodotorula graminis* in the aforementioned S-mandelic acid producing strain led to the *de novo* production of 0.88 g/L of **R-mandelic acid** in shake flask cultures. S-Mandelate synthase HmaS from *A. orientalis* can convert 4-hydroxyphenylpyruvate (4HPP) to 4HMA, but also phenylpyruvate to mandelic acid in *E. coli* (Sun et al., 2011; Li et al., 2016). It was also found that the introduction of codon-optimized *hmaS* from *A. orientalis* in *S. cerevisiae* produced mandelic acid (Reifenrath and Boles, 2018). Mandelic acid production was increased by the replacement of *hmaS* from *A. orientalis* with the corresponding gene from *Nocardia uniformis*. The strategies used for the enhancement of the expression of the aromatic amino acid pathway as well as the deletion of the competing pathways were used to improve the production of mandelic acid. The resulting strain produced 236 mg/L mandelic acid with a mass yield of 1.7% in shake flask cultures (Reifenrath and Boles, 2018). Lukito et al. (2019) developed a whole cell-based cascade biotransformation for the production of (S)-mandelic acid from styrene, L-Phe, glucose, or glycerol. The recombinant *E. coli* strain LZ37 expressing the 6-step enzyme cascades produced 160 mM (S)-mandelic acid from L-Phe with a yield of 80%. Coupling the *E. coli* strain LZ37 with the L-Phe producing *E. coli* strain NST74 enabled the *de novo* production of 63 mM (10 g/L) or 52 mM (8 g/L) (S)-mandelic acid from glycerol and glucose, respectively.

**Cinnamic acid** is used as a flavoring agent and antibacterial compound. The overexpression of *Streptomyces maritimus* L-phenylalanine ammonia lyase gene *pal* in an *E. coli* strain overproducing L-Phe resulted in the production of 6.9 g/L of *trans-*cinnamic acid in a fed-batch fermentation process (Figure 2) (Bang et al., 2018). It was demonstrated that the bacterial phenylalanine ammonia lyase from *Photorhabdus luminescens* was superior to the plant enzyme from *A. thaliana* for the production of *trans-*cinnamic acid in yeast (Gottardi et al., 2017). After optimizing the biosynthetic pathway, the overexpression of *ARO4*^*K*229*L*^*, ARO7*^*G*141*S*^, and codon-optimized *pal* from *P. luminescens*, and deletion of *ARO10* in *S. cerevisiae* CEN.PK113-7D resulted in the production of 37.9 mg/L of *trans-*cinnamic acid (Gottardi et al., 2017).

**Styrene** and its derivatives act as monomers and petroleum-based feedstocks, which is valuable because they can be used as raw materials in industrial processes. They have many uses, including in the manufacture of polystyrenes, plastics, and styrene-butadiene. Styrene can be synthesized from glucose by the co-expression of phenylalanine ammonia lyase and *trans-*cinnamate decarboxylase (Figure 2). Mckenna and Nielsen screened candidate isoenzymes for the two steps from bacterial, yeast, and plant (McKenna and Nielsen, 2011). Finally, the overexpression of *PAL2* from *A. thaliana* and *FDC1* from *S. cerevisiae* in an L-Phe overproducing *E. coli* strain led to the accumulation of 260 mg/L of styrene in shake flask cultures. To avoid the effects of the toxicity of styrene, solvent extraction fermentation was used to improve the amount of styrene produced, which achieved 836 mg/L (McKenna et al., 2015). A similar strategy was also used for achieving styrene production in *E. coli* BL (DE3), which yielded 350 mg/L of styrene in an extraction fermentation process using isopropyl myristate (Liu C. Q. et al., 2018). This titer was achieved by the overexpression of *PAL2* from *A. thaliana, FDC1* from *S. cerevisiae*, native *ppsA, tktA, aroF*, and *pheA*, using a two-plasmid system. An L-Phe overproducing *E. coli* strain overexpressing *S. maritimus pal* and *FDC1* from *S. cerevisiae* was engineered for the production of styrene (Lee et al., 2019). The resulting strain produced 5.3 g/L of styrene in a 5-L fed-batch fermentation process using *in situ* extraction and gas stripping with n-dodecane. The styrene synthetic pathway containing *PAL2* from *A. thaliana* and *FDC1* from *S. cerevisiae* was integrated into the SS9 site of an L-Phe overproducing strain *E. coli* NST 74 to obtain a styrene producing strain (Liang et al., 2020). After optimization of the expression levels of the two genes *PAL2* and *FDC1*, iCREATE strategy for 54 transcription regulator genes was used to improve styrene-tolerance and the production of styrene. The resulting strain produced 3.15 g/L in a 1-L bioreactor fed-batch fermentation using gas stripping strategy with n-dodecane. Although *S. cerevisiae* (McKenna et al., 2014) and *S. lividans* (Fujiwara et al., 2016b) have been used as host strains for styrene production, the titer (about 30 mg/L) is much lower than that obtained using engineered *E. coli*.

**Phenylglycine**, as one of the unnatural amino acids, has been widely used in the synthesis of penicillin, virginiamycin S, pristinamycin I, and the antitumor compound taxol (Liu et al., 2014). Phenylglycine is synthesized from phenylpyruvate via phenylglyoxylate (Figure 2). An artificial biosynthetic pathway, which consists of HmaS (l-4-hydroxymandelate synthase), Hmo (l-4-hydroxymandelate oxidase), and HpgT (l-4-hydroxyphenylglycine transaminase) from *S. coelicolor*, was constructed for the production of L-phenylglycine in *E. coli* (Liu et al., 2014). Deletions of both *tyrB* and *aspC* as well as increasing the copies of both *hmo* and *hpgT* further increased the production of L-phenylglycine to 51.6 mg/gDCW (Liu et al., 2014). A novel L-phenylglycine biosynthetic pathway via the PglE reaction was reported in *Streptomyces pristinaespiralis* (Osipenkov et al., 2018). Zhou et al. (2016) constructed an artificial eight-step biosynthetic pathway containing 10 different enzymes for the production of L-phenylglycine from L-Phe. *E. coli* expressing the pathway produced 34 mM (5.1 g/L) L-phenylglycine with a yield of 85% from L-Phe. An artificial biosynthetic pathway was created for the production of D-phenylglycine in *E. coli* (Muller et al., 2006). The pathway consists of HmaS from *A. orientalis*, Hmo from *S. coelicolor*, and HpgAT from *P. putida*. This pathway was introduced into the L-Phe producing *E. coli* strain KB532 for the *de novo* production of D-phenylglycine. The deletion of both *tyrB* and *aspC* further improved the production of D-phenylglycine, which reached 102 mg/g DCW (Muller et al., 2006). Zhou Y. et al. (2017) created a biosynthetic pathway for the production of D-phenylglycine from mandelic acid, styrene, or L-Phe. The biosynthetic pathway has eight steps catalyzed by nine enzymes: *A. thaliana* PAL2*, Aspergillus niger* phenylacrylic acid decarboxylase (PAD1), styrene monooxygenase (SMO) from *Pseudomonas* sp. VLB120, epoxide hydrolase (SpEH) from *Sphingomonas* sp. HXN-200, alcohol dehydrogenase gene (AlKJ) from *P. putida* GPo1, phenylacetaldehyde dehydrogenase (Aldh) from *E. coli*, (S)-mandelate dehydrogenase (SMDH) from *P. putida* ATCC 12633, D-phenylglycine aminotransferase (DpgAT) from *Pseudomonas stutzeri* ST-201 and glutamate dehydrogenase (GluDH) from *E. coli*. *E. coli* expressing the nine enzymes of the eight-step pathway produced 50 mM D-phenylglycine from 60 mM L-Phe with a yield of 83% using whole-cell catalysis (Zhou Y. et al., 2017).

### Tyrosine Derivatives

**Figure 3:**
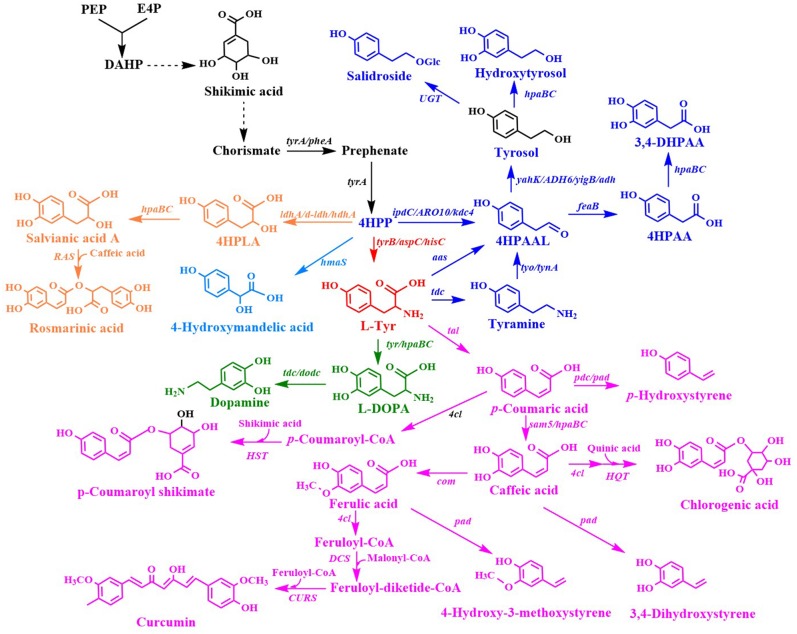
Biosynthesis of L-tyrosine derivatives. 4HPLA, 4-hydroxyphenyllactic acid; 4HPAAL, 4-hydroxyphenylacetaldehyde; 4HPAA, 4-hydroxyphenylacetic acid; *ldhA/d-ldh*, D-lactate dehydrogenase gene; *hdhA*, 2-hydroxyacid dehydrogenase gene; *hpaBC, p*-hydroxyphenylacetate 3-hydroxylase genes; *ipdC/ARO10/kdc*, phenylpyruvate decarboxylase gene; *yahK/ADH6*, alcohol dehydrogenase gene; *UGT*, uridine diphosphate dependent glycosyltransferase gene; *aas*, aromatic aldehyde synthase gene; *RAS*, rosmarinic acid synthase gene; *tyr*, tyrosinase gene; *tdc*, tyrosine decarboxylase gene; *tyo/tynA*, tyramine oxidase gene; *tal*, tyrosine ammonia lyase gene; *dodc*, L-DOPA decarboxylase; *pdc, p*-coumaric acid decarboxylase gene; *4cl*, 4-coumaroyl-coenzyme A ligases gene; *sam5*, 4-coumarate hydroxylase gene; *com*, caffeic acid methyltransferase gene; *HQT*, hydroxycinnamate-CoA quinate transferase gene; *HST*, hydroxycinnamate-CoA shikimate transferase gene; *pad*, phenolic acid decarboxylase gene; *CURS*, curcumin synthase gene.

L-Tyr derivatives were synthesized from 4-hydroxyphenylpyruvate (4HPP) and L-Tyr (Figure 3). Many L-Tyr derivatives were also used as a precursor for the production of a wide range of valuable aromatic compounds with pharmaceutical value (Table 2).

**Table 2:** *De novo* production of tyrosine derivatives by engineered microorganisms.

**Products**	**Host**	**Applications**	**Metabolic engineering strategies**	**Culture conditions**	**Titer**	**Yield**	**References**
4-Hydroxymandelic acid	*E. coli*	Synthesizing aromatic drug and flavors	*ΔptsG, ΔtyrR, ΔpykFA, ΔpheA, ΔtyrB, ΔaspC*, overexpression of *lacUV5* promoter-controlled *aroG^*fbr*^-tyrA^*fbr*^-aroE* and *trc* promoter controlled *ppsA-tktA-glk* on a pMB1 origin plasmid, overexpression of *trc* promoter-controlled *hmo* from *S. coelicolor* on a CloDF13 origin plasmid	5-L bioreactor fed-batch fermentation	15.8 g/L from glucose and xylose		Li et al., 2016
	*S. cerevisiae*		*TRP1, aro4*Δ*::HXT7pr-ARO1, ura3*Δ*::TPI1pr-ARO4^*fbr*^_HXT7pr-coptARO3^*fbr*^, aro10Δaro8Δ pdc5Δ trp2Δ pha2Δ aro9Δ*	Shake-flask fermentation	1 g/L	6.4% g/g	Reifenrath and Boles, 2018
4-Hydroxyphenyllactic acid	*E. coli*	Antifungal compound	Δ*tyrR*::P_T7_-*aroF^*fbr*^-tyrA^*fbr*^ Δacs*::P_T7_-*ldhA*	Shake-flask fermentation	7.6 mM		Koma et al., 2012
Salvianic acid A	*E. coli*	Antioxidant, anti-cancer, anti-inflammatory, and other pharmacological activities	*ΔptsG::*FRT Δ*tyrR::*FRT *ΔpykFA::*FRT *ΔpheA::*FRT, expressing P_lacUV5_- *aroG^*fbr*^-tyrA^*fbr*^-aroE* and P_trc_- *ppsA-tktA-glk* on a pBldgbrick1 (pMB1 origin) plasmid, expressing *hpaBC-dldh^*Y*52*A*^* on a pCDFDuet-1 (pCDF origin) plasmid	500 mL flasks fed-batch fermentation	7.1 g/L,	47% mol/mol	Yao et al., 2013
Rosmarinic acid	*E. coli-E. coli-E. coli*	Antioxidant, anti-microbial anti-tumor, anti-inflammatory, and immune regulation agent	Caffeic acid-producing strain: *ΔpheA, ΔtyrR, lacZ::P_*LtetO*−1−_tyrA^*fbr*^-aroG^*fbr*^ tyrR::P_*LtetO*−1−_tyrA^*fbr*^-aroG^*fbr*^, ΔptsH, ΔptsI, Δcrr, ΔaroE, ΔydiB;* single expressing *Rhodotorula glutinis tal, hpaBC* and *aroE, and rpoA^*mut*^* on a plasmid Salvianic acid A-producing strain: *ΔxylA;* single expressing *P_*proD*_-aroE-aroL-aroA-aroC-tyrA^*fbr*^-aroG^*fbr*^, hpaBC* and *d-ldh^*Y*52*A*^* on a plasmid Rosmarinic acid-producing strain: *ΔpheA, ΔtyrR, lacZ::P_*LtetO*−1−_tyrA^*fbr*^-aroG^*fbr*^ tyrR::P_*LtetO*−1−_tyrA^*fbr*^-aroG^*fbr*^, ΔptsH, ΔptsI, Δcrr, ΔaroE, ΔydiB*. single expressing *Petroselinum crispus 4CL* and *Melissa officinalis RAS* on a plasmid	Coculture in shake flasks	172 mg/L		Li et al., 2019c
Tyrosol	*E. coli*	Antioxidant, anti-cancer and anti-inflammatory activies	*ΔfeaB*. Expressing *Micrococcus luteus tyo* and *Papaver somniferum tdc* on a pSC101 origin plasmid	Shake-flask fermentation	0.5 mM		Satoh et al., 2012
	*E. coli*		Δ*tyrR*::P_T7_-*aroF^*fbr*^-tyrA^*fbr*^ ΔmtlA::*P_T7_-*ipdC Δacs::*P_T7_-*yahK ΔfeaB ΔpheA*	Shake-flask fermentation	8.3 mM (1.15 g/L)		Koma et al., 2012
	*E. coli* MG1665		*ΔfeaB ΔpykFA ΔtyrR ΔpheA*. Plasmid-expressing *S. cerevisiae ARO10* on a pBR322 origin plasmid, plasmid-expressing *Ltet* promoter-controlled t*yrA^*fbr*^-aroG^*fbr*^-ppsA, lacUV5* promoter-controlled *tktA-aroE* and *lacUV5* promoter-controlled *aroD-aroB* on a p15A origin plasmid	Shake-flask fermentation	926.9 mg/L		Bai et al., 2014
	*E. coli* BL21 (DE3)		*ΔfeaB ΔpheA*. Plasmid-expressing *S. cerevisiae ARO10* on a RSF1030 origin plasmid	Shake-flask fermentation	4.15 mM		Xue et al., 2017
	*E. coli* BL21 (DE3)		Δ*tyrR ΔfeaB ΔpheA*. Plasmid-expressing *Petroselinum crispum* aromatic aldehyde synthase gene on a CloDE13 origin plasmid	Shake-flask fermentation	539.4 mg/L		Chung et al., 2017
	*E. coli* BW25113		*ΔfeaB*, Plasmid-expressing *S. cerevisiae ARO10* and *ADH6* on a ColE origin plasmid (pZS12-luc), plasmid-expressing P_LlacO1_ promoter-controlled *tyrA^*fbr*^-ppsA-tktA-aroG^*fbr*^* on a p15A origin plasmid	Shake-flask fermentation	550 mg/L		Li X. et al., 2018
	*E. coli* BW25113		*ΔptsG ΔtyrR ΔpykA ΔpykF ΔpheA, Δmao-paa* cluster::P_lacUV5_-*aroG^*fbr*^-tyrA^*fbr*^-aroE, ΔfeaB::*FRT, *ΔmanZ:*FRT. Plasmid-expressing the codon-optimized *kdc4* from *Pichia pastoris* GS115 on a pTrc99A plasmid	Shake-flask fermentation	1.47 g/L from xylose		Liu X. et al., 2018
Hydroxytyrosol	*E. coli* BL21 (DE3)	Antioxidant, anti-cancer and anti-inflammatory activities	Δ*tyrR ΔfeaB ΔpheA*, plasmid-expressing *P. crispum* aromatic aldehyde synthase gene and *E. coli hpaBC* on a CloDE13 origin plasmid	Shake-flask fermentation	208 mg/L		Chung et al., 2017
	*E. coli* BW25113		*ΔfeaB* Plasmid-expressing *S. cerevisiae ARO10* and *ADH6* on a ColE origin plasmid (pZS12-luc), plasmid-expressing the P_LlacO1_ promoter-controlled *tyrA^*fbr*^-ppsA-tktA-aroG^*fbr*^* and *hpaBC* on a p15A origin plasmid	Shake-flask fermentation	647 mg/L		Li X. et al., 2018
	*E. coli* BL21 (DE3)		Δ*tyrR ΔpheA ΔfeaB* Expressing *aroG^*fbr*^-tyrA^*fbr*^* on a p15 A origin plasmid, expressing *E. coli* W *hpaBC* on pET-duet, expressing tyrosine decarboxylase (TDC) from *P. somniferum* and tyrosine oxidase (TYO) from *M. luteus*	Shake-flask fermentation	268.3 mg/L		Choo et al., 2018
	*E. coli*		*ΔfeaB* Expressing *hpaBC^*S*210*T*/*A*211*M*/*Q*212*G*^*, tyrosine decarboxylase gene (*tdc*) from *Enterococcus faecalis* and tyramine oxidase gene (*tyo*) from *M. luteus*	Shake-flask fermentation with feeding tyrosine by three times	1890 mg/L		Chen et al., 2019
Salidroside	*E. coli* MG1665	Anti-aging, anoxia resistance, and anti-inflammation activities, as well as cardiovascular disease prevention, anti-cancer, nerve, and brain cell protection properties	*ΔfeaB ΔpykFA ΔtyrR ΔpheA*. Expressing the *trc* promoter-controlled *S. cerevisiae ARO10* and the *trc* promoter-controlled *Rhodiola sachalinensis UGT73B6* on a pBR322 origin plasmid. Expressing the *Ltet* promoter-controlled t*yrA^*fbr*^-aroG^*fbr*^-ppsA*, the *lacUV5* promoter-controlled *tktA-aroE* and the *lacUV5* promoter-controlled *aroD-aroB* on a p15A origin plasmid	Shake-flask fermentation	56.9 mg/L		Bai et al., 2014
	*E. coli* BL21 (DE3)		Δ*tyrR ΔfeaB ΔpheA*. Expressing *P. crispum* aromatic aldehyde synthase gene on a CloDE13 origin plasmid. Expressing *A. thaliana UGT85A1* on a f1 origin plasmid	Shake-flask fermentation	287.9 mg/L		Chung et al., 2017
	*E. coli- E. coli*		AD strain: BW25113 *ΔptsG::ΔtyrR::ΔpykA:: pykF::ΔpheA::*FRT, *Δmao-paa* cluster::P_lacUV5_-*aroG^*fbr*^-tyrA^*fbr*^-aroE, ΔfeaB::*FRT, *ΔmanZ:*FRT; GD strain: BL21(DE3) *ΔushA:*FRT Δ*tyrA::pgm-galU, ΔxylA:*FRT. Expressing the codon-optimized *UGT85A1* from *A. thaliana* on a CloDF13 origin plasmid	5-L bioreactor fed-batch	6.03 g/L		Liu X. et al., 2018
Tyrosol/ salidroside	*S. cerevisiae* BY4742		Integrating with *URA3* selectable marker and P_PGK1_-*ARO7^*G*141*S*^*-T_ADH1_-P_TDH3_-*ARO4^*K*229*L*^*-T_TEF1_- P_TEF1_-*AroL*-T_CYC1_ cassettes into *YPRCΔ15* DNA site. Integrating with *LEU2* selectable marker and P_PGK1_-*AtUGT85A1*_syn_-T_ADH1_-P_TDH3_-*PcAAS_*syn*_*- T_TEF1_ cassettes into *YJR056C* DNA site	5-L bioreactor fed-batch fermentation	732.5 mg/L salidroside and 1394.6 mg/L tyrosol		Jiang et al., 2018
4-Hydroxyphenylacetic acid	*E. coli*	Synthesizing drugs, agrochemicals, biochemicals	Δ*tyrR*::P_T7_-aroF^fbr^-pheA^fbr^, *ΔmtlA*::P_T7_-*ipdC, Δacs*::P_T7_-*feaB, ΔyahK, ΔpheA*	Shake-flask fermentation	6.1 mM (928.1 mg/L)		Koma et al., 2012
	*E. coli* DOPA-30N (L-DOPA producer)		Expressing the TIGR-mediated gene cluster of the evolved *ARO10_6*D*5_* and *feaB_2*E*1_* under the control of the Esa-P_esaR_ activation system	2-L bioreactor fed-batch fermentation	17.39 g/L		Shen et al., 2019
*p*-Coumaric acid	*E. coli* BL21	Precursor of flavonoids, polyphenols, and polyketides	Expressing tyrosine ammonia lyase gene *tal* from *Saccharothrix espanaensis* in a tyrosine-overproducing *E. coli* expressing *E. coli aroG^*fbe*^* and *tyrA^*fbr*^* on a p15A origin plasmid (pACYDuet-1)	Shake-flask fermentation	974 mg/L		Kang et al., 2012
	*S. cerevisiae* CEN.PK102-5B		MATa, *aro10Δ pdc5Δ*, pX2:: P*_*TEF*1_*-*TAL*-T*_*ADH*1_*, pX-4::P*_*TEF*1_*-*ARO7^*fb*^*^r^-T*_*ADH*1_*, P*_*PGK*1_*- *ARO4^*fbr*^*-T*_*CYC*1_*, pX-3::P*_*PGK*1_*-*aroL*-T*_*CYC*1_*	Fed-batch fermentation	1.93 g/L		Rodriguez et al., 2015
	*S. cerevisiae* S288C		*MATα, aro10Δ pdc5Δtat1Δ*, pX-2:: P*_*TEF*1_*-*TAL*-T*_*ADH*1_*, pX-4::P*_*TEF*1_*-*ARO7^*fb*^*^r^-T*_*ADH*1_*, P*_*PGK*1_*- *ARO4^*fbr*^*-T*_*CYC*1_*, pX-3::P*_*PGK*1_*-*aroL*-T*_*CYC*1_*	Fed-batch fermentation	3.56 g/L		Rodriguez et al., 2017
	*S. cerevisiae* strain CEN.PK113-5D-derivative IMX581		Introducing a phosphoketalose-based pathway and the replacement of promoters	Fed-batch fermentation	12.5 g/L		Liu Q. et al., 2019
Caffeic acid	*E. coli* BL21	Pharmaceuticals, esters, and polymers	Expressing *S. espanaensis* tyrosine ammonia lyase gene *tal* and 4-coumarate hydroxylase gene *sam5* in a tyrosine-overproducing *E. coli* expressing *E. coli aroG^*fbe*^* and *tyrA^*fbr*^* on a p15A origin plasmid (pACYDuet-1)	Shake-flask fermentation	150 mg/L		Kang et al., 2012
	*E. coli* BW25113		Expressing *Rhodobacter capsulatus* tyrosine ammonia lyase gene *tal* and *E. coli* 4-hydroxyphenylacetate 3-hydroxylase gene *hpaBC* on a pZE12-luc plasmid. Expressing the P_LlacO1_-controlled *tyrA^*fbr*^-ppsA-tktA-aroG^*fbr*^* on a p15A origin plasmid	Shake-flask fermentation	50.2 mg/L		Lin and Yan, 2012
	*E. coli* ATCC 31884 (L-Phe over-producer)		*ΔpheLAΔtyrA* Expressing the P_LlacO1_-controlled *R. glutinis* tyrosine ammonia lyase gene *tal* and the P_LlacO1_-controlled *E. coli* 4-hydroxyphenylacetate 3-hydroxylase gene *hpaBC* on a ColE1 origin plasmid. Expressing the P_LlacO1_-controlled *tyrA^*fbr*^-ppsA-tktA-aroG^*fbr*^* on a p15A origin plasmid	Shake-flask fermentation	766.68 g/L		Huang et al., 2013
	*E. coli* MG1655		*ΔpheLA ΔtyrR, lacZ::P_*LtetO*−1_-tyrA^*fbr*^aroG^*fbr*^ ΔtyrR::P_*LtetO*−1_-tyrA^*fbr*^aroG^*fbr*^*, pHACM-rpoA14. Expressing the codon-optimized *R. glutinis tal* on a pBR322 origin plasmid. Expressing the codon-optimized *S. espanaensis* 4-coumarate 3-hydroxylase gene on a CloDF13 origin plasmid	Shake-flask fermentation	106 mg/L		Zhang and Stephanopoulos, 2013
	*S. cerevisiae*		Expressing *Rhodosporidium toruloides TAL* on a plasmid (pRS416), *Pseudomonas aeruginosa hpaB* on a plasmid (pRS415), and *Salmonella enterica hpaC* on a plasmid (pRS413)	Shake-flask fermentation	289.4 mg/L		Liu L. et al., 2019
Ferulic acid	*E. coli* BL21	Cosmetics and pharmaceuticals	Expressing *S. espanaensis* tyrosine ammonia lyase gene *tal*, 4-coumarate hydroxylase gene *sam5* and *A. thaliana* caffeic acid methyltransferase gene *com* in a tyrosine-overproducing *E. coli* expressing *E. coli aroG^*fbe*^* and *tyrA^*fbr*^* on a p15A origin plasmid	2-L bioreactor fermentation	196 mg/L		Kang et al., 2012
*p*-Hydroxystyrene	*E. coli* ATCC 31884 (NST 74) (L-Phe overproducer)	Polymers	Expressing *R. glutinis pal* on a pKK2233 plasmid. Expressing *Lactobacillus plantarum pdc* on a pKSM715 plasmid	Fed-batch fermentation	0.4 g/L		Qi et al., 2007
	*E. coli*	Synthesizing plastics and rubbers	Δ*tyrR::tyrA^*fbr*^-aroG^*fbr*^*. Expressing *S. espanaensis tal* and phenolic acid decarboxylase gene *pad* from *Bacillus amyloliquefaciens* on pET-28a (+) plasmid	Shake-flask fermentation	355 mg/L		Kang et al., 2012
	*Streptomyce lividans*		Expressing *pad* from *S. Sviceus* and *tal* from *Rhodobacter sphaeroids*. Expressing endoglucanase gene *tfu0901* from *Thermobifida fusca* YX	Shake-flask fermentation	250 mg/L from cellulose, 360 mg/L from glucose		Noda et al., 2015
	*Streptomyce mobaraense*		Expressing *tal* from *Rhodobacter sphaeroids*	Shake-flask fermentation	273 mg/L		Fujiwara et al., 2016a
	*P. putida* S12		*Δfcs*. Expressing the *pal* and *pdc* genes under the control of the salicylate-inducable *NagR/pNagAa* promoter and *tac* RBS	3-L two-phase water-decanol fed-batch fermentation	147 mM (17.6 g/L)		Verhoef et al., 2009
3,4-Dihydroxystyrene	*E. coli*		Δ*tyrR::tyrA^*fbr*^-aroG^*fbr*^*. Expressing *tal* and *sam5* from *S. espanaensis*, and *pad* from *B. amyloliquefaciens* on pET-28a (+) plasmid	Shake-flask fermentation	63 mg/L		Kang et al., 2015
4-Hydorxy-3-methoxystyrene	*E. coli*	Synthesizing plastics and rubbers	Δ*tyrR::tyrA^*fbr*^-aroG^*fbr*^*. Expressing *tal* and *sam5* from *S. espanaensis, A. thaliana com*, and *B. amyloliquefaciens pad* from on pET-28a (+) plasmid	Shake-flask fermentation	64 mg/L		Kang et al., 2015
Chlorogenic acid	*E. coli*-*E. coli*	Antioxidant, anti-bacterial, antiviral, antitumor, and anti-inflammatory agent as well as drugs	Caffeic acid-producing strain: Δ*tyrR ΔPheA*, expressing *S. espanaensis TAL, aroG* and *tyrA* on a p15A origin plasmid (pACYCDuet), and *hpaBC* on a f1 origin plasmid (pETDUet) Chlorogenic acid-producing strain: Δ*aroD*, expressing *N. tabacum HQT* and *Oryza sativa 4CL* on a p15A origin plasmid (pACYCDuet), and native *ydiB* on a f1 origin plasmid (pETDUet)	Coculture in shake flasks	78 mg/L		Cha et al., 2014
*p*-Coumaroyl shikimate	*E. coli*	Antioxidant, anti-bacterial, antiviral, antitumor, and anti-inflammatory agent as well as drugs	ΔtyrR ΔPheAΔaroL Expressing *S. espanaensis TAL, aroG* and *tyrA* on a p15A origin plasmid (pACYCDuet), and *N. tabacum HST* and *O. sativa 4CL* on a CloDE13 origin plasmid (pCDFDuet)	Shake-flask fermentation	236 mg/L		Cha et al., 2014
Curcumin	*E. coli*	Antioxidant, anti-bacterial, antiviral, antitumor, and anti- inflammatory agent as well as treatment of Parkinson's and Alzhemier's disease	Δ*tyrR::tyrA^*fbr*^-aroG^*fbr*^* Chromosomal expressing *S. espanaensis tal, S. espanaensis sam5, A. thaliana com, Nicotiana tabacum 4cl, Curcuma longa DCS*, and *C. longa CURS2*. MAGE was used to optimizate the expression ratios of the six enzymes	Shake-flask fermentation	3.8 mg/L		Kang et al., 2018
L-DOPA	*E. coli*	Treating Parkinson's disease	Expressing *Streptomyces castaneoglobisporus* tyrosinase gene on a pET-23a plasmid. Expressing *tyrA^*fbr*^-aroG^*fbr*^-tktA-ppsA* on a pCOLADuet-1 plasmid	Shake-flask fermentation	293 mg/L		Nakagawa et al., 2011
L-DOPA	*E. coli*		*ΔptsI, ΔptsH, Δcrr::kan, ΔlacI, ΔlacZ::loxP*, PgalP::Ptrc, Δ*tyrR*. Expressing the *trc* promoter-controlled *E. coli* W *hpaBC*, the *trc* promoter-controlled *Zymomonas mobilis tyrC* and the native *pheA_*CM*_* on a pTrc99A plasmid. Expressing the *lacUV5* promoter-controlled *aroG^*fbr*^* and *tktA* under the control of its native paomoter on a p15A origin plasmid	Batch fermentation	1.5 g/L		Munoz et al., 2011
L-DOPA	*E. coli*		MAGE strain, Δ*tyrR*, Δ*csrA*, Δ*ptsHI*, Δ*crr*, P_37_-*galP*-P_37_-*glk*, Δ*zwf*, Δ*pheLA*, attP_P21_::7P37-*tyrA^*fbr*^-tyrB-hpaBC* fusion protein chimera, *aroG::aroG^*Asp*146*Asn*^*	Fed-batch fermentation	8.67 g/L		Wei et al., 2016
L-DOPA	*E. coli*		Δ*tyrR,ΔpykF, ΔserA, aroG::aroG^*fbr*^*, expressing *E. coli* W *hpaBC* on a pBR322 origin plasmid. Expressing the *lacUV5* promoter-controlled *tyrB-tyrA^*fbr*^-aroC* and the *lacUV5* promoter-controlled *aroA-aroL* on a p15A origin plasmid	Fed-batch fermentation	12.5 g/L		Das et al., 2018
Dopamine	*E. coli*		Expressing *S. castaneoglobisporus* tyrosinase gene and *P. putida* KT2440 L-DOPA decarboxylase gene *dodc* on a pET-23a plasmid. Expressing *tyrA^*fbr*^-aroG^*fbr*^-tktA-ppsA* on a pCOLADuet-1 plasmid	Shake-flask fermentation	260 mg/L		Nakagawa et al., 2011
	*E. coli*		Δ*tyrR*, Δ*crr*, Δ*ptsG*, ΔpheA Δ*pyk* Expressing *hpaB^*G*295*R*^-hpaC* on a plasmid (pRSFDuet-1), *P_*T*7_-galP-P_*T*7_-glk-P_*T*7_-ppsA-P_*T*7_-tktA* on a plasmid (pETM6), *aroG^*fbr*^-tyrA^*fbr*^* on a plasmid (pCDFDuet-1)	5 L bioreactor fed-batch fermentation	25.53 g/L		Fordjour et al., 2019

**4HMA** serves as a building block for the synthesis of aromatic drugs and flavors. The biosynthetic pathway for 4HMA production has been successfully established in *E. coli* and *S. cerevisiae* (Li et al., 2016; Reifenrath and Boles, 2018). The overexpression of the codon-optimized *hmaS* from *A. orientalis* in the tyrosine-producing strain, *E. coli* BKT5, resulted in the formation of 4HMA (Figure 3) (Li et al., 2016). After optimizing the expression of *hmaS* by fine-tuning four promoters of different strengths which were combined with three plasmids with different copy numbers, and blocking the competing pathway by deleting *tyrB* and *aspC*, 4HMA production was improved. The resulting strain produced 15.8 g/L 4HMA from a mixture of glucose and xylose in a 5-L bioreactor fed-batch fermentation process. It was also found that the introduction of the codon-optimized *hmaS* from *A. orientalis* in *S. cerevisiae* produced 4HMA (Reifenrath and Boles, 2018). The production of 4HMA was increased by the replacement of *hmaS* from *A. orientalis* with the corresponding gene from *N. uniformis*. The strategies used for the enhancement of the expression of the aromatic amino acid pathway as well as the deletion of the competing pathways were used to improve the production of 4HMA. The strain exhibiting the best performance produced 1 g/L of 4HMA with a mass yield of 6.4% in shake flask cultures (Reifenrath and Boles, 2018).

**4-Hydroxyphenyllactic acid** (4HPLA) is known to be an antifungal compound. The replacement of *acs* with *C. necator* JCM20644 *ldhA* under the control of the T7 promoter in the L-Tyr producing *E. coli* strain resulted in the production of 7.6 mM 4HPLA in shake flask cultures (Figure 3) (Koma et al., 2012).

**Salvianic acid A** (3,4-dihydroxyphenllactic acid, also known as danshensu), is a naturally occurring plant polyphenolic acid that is recognized for its superior antioxidant activities. It has a variety of other pharmacological activities, including improved cerebral blood flow, inhibition of platelet activation and arterial thrombosis, as well as anti-cancer and anti-inflammatory activities. Yao et al. developed an artificial biosynthetic pathway for salvianic acid A production in *E. coli* (Yao et al., 2013). 4-Hydroxyphenylpyruvate was converted to salvianic acid A via D-lactate dehydrogenase (encoded by *d-ldh* from *Lactobacillus pentosus*) and the hydroxylase complex (encoded by *hpaBC* from *E. coli*) (Figure 3). After optimizing the pathway using a modular engineering approach and deleting the genes involved in regulatory and competing pathways, the metabolically engineered *E. coli* strain produced 7.1 g/L of salvianic acid A with a yield of 0.47 mol/mol glucose. To overcome the drawbacks caused by plasmid system, the salvianic acid A biosynthetic pathway was integrated into the chromosomal of the L-Tyr producing *E. coli* strain BAK5 to obtain a plasmid-free strain for the production of salvianic acid A (Zhou L. et al., 2017). The final strain BKD13 produced 5.6 g/L salvianic acid A by fed-batch fermentation. A cell-free system was established for the production of salvianic acid A from phenylpyruvate. The cell-free system contained D-mandelate dehydrogenase from *Thermococcus barophilus*, phenylalanine 4-hydroxylase from *Thermomonospora curvata*, and Hydroxyphenylacetate 3-hydroxylase from *Sulfobacillus acidophilus* TPY. The resulting cell-free system produced 392 mM of salvianic acid A with a yield of 85% (Li et al., 2019a).

**Rosmarinic acid** is a plant-derived natural compound belong to the family of polyphenolic compounds. It is an ester of caffeic acid and salvianic acid A. It has diverse important nutraceutical and pharmaceutical values, including antioxidant, anti-inflammatory, anti-microbial, anti-tumor and anti-viral, and immune regulation activities. After comparing pathway enzymes, Bloch and Schmidt-Dannert (2014) constructed an artificial biosynthetic pathway of rosmarinic acid (Figure 3), which consisted of 2-hydroxyacid dehydrogenase (HdhA) from *Lactobacillus delbrueckii*, 4-hydroxyphenylacetate 3-hydroxylase complex (HpaBC) from *E. coli W*, Tal from *Rhodobacter sphaeroides*, 4-coumarate-CoA ligase 2 (4CL2) from *A. thaliana* and rosmarinic acid synthase from *Melissa officinalis*. The introduction of this pathway into *E. coli* by using three plasmids resulted in the *de novo* production of rosmarinic acid. Jiang et al. (2016) established another biosynthetic pathway of rosmarinic acid using a different set of enzymes. The recombinant *E. coli* strain harboring this pathway produced 130 mg/L rosmarinic acid from caffeic acid using whole-cell biocatalysis. The biosynthetic pathway of rosmarinic acid from 4-hydroxyphenylpyruvate is a diverging and converging pathway. Modular co-culture engineering with three *E. coli* strains was used to balance the different pathway modules for the production of rosmarinic acid (Li et al., 2019c). The three-strain co-culture produced 172 mg/L rosmarinic acid, exhibiting 38-fold improvement over the mono-culture. Yan et al. (2019) established a cell-free system with ATP and CoA double regeneration for the production of rosmarinic acid, which reached 320.04 mg/L/h.

**Tyrosol** and hydroxytyrosol are two major ingredients in olive oil, and both are considered bioactive ingredients that have various biological activities. It has been reported that tyrosol plays an important role in the prevention of cardiovascular diseases, osteopenia, melanin pigmentation, as well as anti-inflammatory responses (Kim et al., 2018). In addition, tyrosol can lower the risk of developing Alzheimer's disease (Kim et al., 2018). Three biosynthetic pathways have been developed for tyrosol production (Figure 3). In the first pathway, tyrosine is converted into tyramine by tyrosine decarboxylase (TDC), which is followed by the further consecutive transformation of tyramine into tyrosol by tyramine oxidase (TYO) and ADH (Satoh et al., 2012). The second pathway is known as the yeast Ehrlich pathway, wherein 4HPP is decarboxylated to 4-hydroxyphenylacetaldehyde (4HPAAL), which is acted on by the endogenous enzyme ADH (Hazelwood et al., 2008; Bai et al., 2014; Xue et al., 2017). In the third pathway, tyrosine is directly converted to 4HPAAL by aromatic aldehyde synthase (AAS), and then converted to tyrosol by ADH (Chung et al., 2017; Jiang et al., 2018). The plasmid-mediated overexpression of the *tyo* gene from *Micrococcus luteus* and tyrosine decarboxylase gene from *Papaver somniferum* in the *feaB*-knockout mutant of *E. coli* BW25113 resulted in the production of 0.50 mM of tyrosol (Satoh et al., 2012) in shake flask cultures. The yeast Ehrlich pathway for tyrosol production was established by the overexpression of indol-3-pyruvate/phenylpyruvate decarboxylase gene *ipdC* from *A. brasilense* NBRC102289 (Koma et al., 2012). The co-overexpression of *A. brasilense ipdC* and *E. coli yahK*, as well as the deletions of *feaB* and *pheA* in a Tyr-overproducing *E. coli* strain resulted in the production of 8.3 mM (1.15 g/L) tyrosol in shake flask cultures (Koma et al., 2012). The yeast Ehrlich pathway for tyrosol production was also established by the overexpression of *S. cerevisiae* pyruvate decarboxylase gene *ARO10* (Bai et al., 2014). A combination of the knock out of the competitive pathways and negative regulation, along with the enhanced expression of the pathway genes enhanced the level of tyrosol produced to 926.9 mg/L. In another study, *ARO10* from *S. cerevisiae* was overexpressed in the *pheA/feaB* double knockouts of *E. coli* BL21 (DE3), which resulted in the synthesis of 4.15 mM (573.4 mg/L) tyrosol from glucose during 48-h shake flask culture (Xue et al., 2017). Recently, it was demonstrated that the overexpression of the *S. cerevisiae* ADH gene *ADH6* further increased tyrosol production (Li X. et al., 2018). The resulting *E. coli* strain containing the yeast Ehrlich pathway produced 1,469 mg/L of tyrosol from tyrosine and 550 mg/L of tyrosol from a simple carbon source, such as glucose. The overexpression of *E. coli hpaBC* in the tyrosol-overproducing strain led to the production of 647 mg/L **hydroxytyrosol** from a simple carbon source, such as glucose (Figure 3) (Li X. et al., 2018). In another recent study, the *kdc4* gene from *P. pastoris* GS115 was more efficient for the biosynthesis of tyrosol than the previously reported *ARO10* gene from *S. cerevisiae* (Liu X. et al., 2018). After the deletion of genes involved in regulatory and competing pathways, the resulting *E. coli* strain produced 1.47 g/L tyrosol from xylose in shake flask cultures, which is the highest titer produced so far. An AAS-derived pathway was first established for tyrosol and hydroxytyrosol production by the overexpression of the *aas* gene from *Petroselinum crispum* (Chung et al., 2017). The three different *aas* genes from *A. thaliana, Petunia hybrid*, and *P. crispum* were compared, and it was found that the *P. crispum aas* gene was most suitable for the production of tyrosol and hydroxytyrosol in *E. coli*. The deletion of *tyrR, pheA*, and *feaB* further increased tyrosol production. The overexpression of the *aas* gene from *P. crispum* in the *tyrR*/*pheA*/*feaB* knockouts of *E. coli* BL21 (DE3) resulted in the production of 539.4 mg/L of tyrosol in shake flask cultures (Chung et al., 2017). The authors also found that the titer of the product generated using the AAS-derived pathway was higher than that generated using the TDC-TYO-derived pathway. Hydroxytyrosol can be synthesized from tyrosol by hydroxylation (Figure 3). Chung et al. (2017) compared *E. coli hpaBC* and *Saccharothrix espanaensis sam5* and found that the co-overexpression of *E. coli hpaBC* and *P. crispum aas* in *E. coli* produced greater levels of hydroxytyrosol. The plasmid overexpression of *E. coli hpaBC* and *P. crispum aas* in the tyrosol-overproducing strain resulted in the production of 208 mg/L of hydroxytyrosol in shake flask cultures (Chung et al., 2017). Judging from the reported titers of tyrosol, the yeast Ehrlich pathway has the highest efficiency among the three pathways, subsequent to the AAS-derived pathway. **Salidroside** is a glucoside of tyrosol and one of the major ingredients of the medicinal herb *Rhodiola*. It has various biological properties, including anti-aging, anoxia resistance, and anti-inflammation activities, as well as cardiovascular disease prevention, anti-cancer, nerve protection, and brain cell protection properties (Bai et al., 2014). It can be synthesized from tyrosol by a uridine diphosphate dependent glycosyltransferase (UGT) (Figure 3). Salidroside was first produced by introducing the glycosyltransferase UGT73B6 from *Rhodiola sachalinensis* into the tyrosol-overproducing *E. coli* strain (Bai et al., 2014). The resulting *E. coli* strain produced 56.9 mg/L of salidroside in shake flask cultures. Twelve UGTs from *A. thaliana* were screened, and UGT85A1 was found to be the most suitable enzyme for the production of salidroside from tyrosol (Chung et al., 2017). The overexpression of *A. thaliana UGT85A1* in the tyrosol-overproducing *E. coli* strain with the AAS-derived pathway resulted in the production of 287.9 mg/L salidroside in shake flask cultures. The results of the two studies described above showed that most of the tyrosol was not glycosylated into salidroside (Bai et al., 2014; Chung et al., 2017). To overcome the drawbacks observed in *E. coli* monocultures, a syntrophic *E. coli-E. coli* co-culture system was developed for salidroside production (Liu X. et al., 2018). The co-culture system composed of the aglycone (AG) and the glycoside (GD) strains, which convergently accommodated the biosynthetic pathways for the production of tyrosol and salidroside, respectively. The co-culture of the AG and DG strains resulted in the production of 6.03 g/L salidroside in a 5-L bioreactor fed-batch fermentation process (Liu X. et al., 2018). Tyrosol is a native metabolite of yeast that is derived from endogenous Ehrlich pathway. Thus, *S. cerevisiae* has been successfully used as a host strain for tyrosol production. An AAS-derived pathway has been successfully introduced into *S. cerevisiae* for tyrosol and salidroside production (Jiang et al., 2018). Plasmid overexpressing of *aas* from *P. crispum* in *S. cerevisiae* improved the production tyrosol to 398.9 mg/L from 170.8 mg/L. Chromosomal overexpression of *P. crispum aas, A. thaliana UGT85A1* as well as the enhancement of the expressions of *ARO4*^*K*229*L*^*, ARO7*^*G*141*S*^, and *AROL* resulted in the production of tyrosol and salidroside to 1394.6 and 732.5 mg/L, respectively (Jiang et al., 2018). An AAS-derived pathway containing *P. crispum* Aas and *E. coli* Adh was overexpressed for the production of tyrosol in *S. cerevisiae* (Guo et al., 2019). The replacement of *PDC1* with *E. coli tyr* mutant *tyrA*^*M*53*I*/*A*354*V*^ further improved the production of tyrosol by 440 times. The introduction of tyrosine decarboxylase from *P. somniferum*, tyrosine oxidase from *M. luteus*, and 4-hydroxyphenylacetate 3-monooxygenase from *E. coli* in a tyrosine-overproducing *E. coli* strain resulted in the production of 268.3 mg/L of hydroxytyrosol (Choo et al., 2018). A HpaBC mutant (HpaBC^S210T/A211M/Q212G^) active on both tyrosol and tyramine was obtained by directed divergent evolution (Chen et al., 2019). Then, they constructed a multiple pathway network to convert tyrosine to hydroxytyrosol using the same set of enzymes. Overexpression of the *hpaBC* mutant, tyrosine decarboxylase gene (*tdc*) from *Enterococcus faecalis* and tyramine oxidase gene (*tyo*) from *M. luteus* in the *feaB* deletion *E. coli* strain enabled the production of hydroxytyrosol of 1,890 mg/L (Chen et al., 2019). A whole-cell catalytic method was developed for the production of hydroxytyrosol from L-DOPA (Li et al., 2019b). Aromatic amino acid aminotransferase (TyrB) from *E. coli* and aldehyde reductase (YahK) from *E. coli*, a-keto acid decarboxylase (Kdc) from *P. mirabilis* JN458, and L-glutamate dehydrogenase (Gdh) were co-expressed in *E. coli* to catalyze the production of hydroxytyrosol from L-DOPA. The yield of hydroxytyrosol reached 36.33 mM (5.59 g/L) using whole-cell catalysis of the recombinant *E. coli* (Li et al., 2019b).

The production of **4HPAA** was achieved by the overexpression of endogenous *feaB* and *ipdC* from *A. brasilense* NBRC102289 in *E. coli*, using the Ehrlich pathway intermediates 4-hydroxyphenylacetaldehyde as precursors (Koma et al., 2012). The overexpression of *A. brasilense ipdC* and *E. coli feaB*, as well as the deletion of *yahK*/*pheA* in the Tyr-overproducing *E. coli* strain resulted in the production of 6.1 mM (928.1 mg/L) of 2-phenylacetic acid in shake flask cultures (Figure 3) (Koma et al., 2012). Our lab constructed an *E. coli* strain for the production of 4HPAA by applying a combinatorial strategy of directed evolution of pathway enzymes and quorum-sensing (QS)-based dynamic regulation of the pathway. The resulting strain produced 17.39 g/L 4HPAA in 2-L bioreactor fed-batch fermentation (Shen et al., 2019). The 4HPAA titer is the highest value reported to date.

Hydroxycinnamic acids are a class of hydroxylate aromatic acids synthesized via the phenylpropanoid pathway. They mainly include cinnamic acid, caffeic acid, *p*-coumaric acid, and ferulic acid. The biotechnological production of ***p*-coumaric acid** was first achieved in *S. cerevisiae* from phenylalanine by phenylalanine ammonia lyase (PAL)-catalyzed formation of cinnamic acid and sequential hydroxylation of cinnamic acid via the cytochrome P450-dependent enzyme 4-cinnamic acid hydroxylase (C4H) (Ro and Douglas, 2004). However, the lower expression of C4H led to the production of an extremely low level of *p*-coumaric acid. To improve *p*-coumaric acid production, a more straightforward route that employed tyrosine as the direct precursor was established in *E. coli*. The overexpression of the tyrosine ammonia lyase gene *tal* from *S. espanaensis* in a tyrosine-overproducing *E. coli* strain resulted in the production of 974 mg/L of *p*-coumaric acid in shake flask cultures (Figure 3) (Kang et al., 2012). The overexpression of the heterologous biosynthetic pathway of caffeic acid (including *S. espanaensis* tyrosine ammonia lyase gene *tal* and 4-coumarate hydroxylase gene *sam5*) or ferulic acid (including tyrosine ammonia lyase gene *tal*, 4-coumarate hydroxylase gene *sam5* from *S. espanaensis*, and caffeic acid methyltransferase gene *com* from *A. thaliana*) (Figure 3) led to the production of 150 mg/L of **caffeic acid** or 196 mg/L **ferulic acid**, respectively (Kang et al., 2012). This pathway was also introduced in *S. cerevisiae* to enable the production of 1.9 g/L of *p*-coumaric acid in a deep-well plate fed-batch fermentation process (Rodriguez et al., 2015). This titer was obtained by the inactivation of phenylpyruvate decarboxylase and pyruvate decarboxylase, in conjunction with the overexpression of genes encoding for feedback-resistant DAHP synthase, chorismate mutase, and shikimate kinase (Rodriguez et al., 2015). Then, this research group further enhanced *p*-coumaric acid titer to 3.56 g/L by deleting the amino acid transporter gene *tat1* (Rodriguez et al., 2017). Recently, this group further enhanced the production of *p*-coumaric acid to 12.5 g/L by introducing a phosphoketalose-based pathway to divert glycolytic flux toward erythrose 4-phosphate synthesis, and optimizing carbon distribution between glycolysis and the aromatic amino acid biosynthetic pathway by promoter replacement (Liu Q. et al., 2019). The *p*-coumaric acid titer is the highest value reported to date. This pathway has also been introduced into *P. putida* KT2440 (Calero et al., 2016) and *Synechocystis* PCC 6803 (Xue et al., 2014; Tantong et al., 2018) to achieve the production of *p*-coumaric acid. However, the *p*-coumaric acid titer obtained with these engineered microorganisms was lower than that obtained with *S. cerevisiae*. **Caffeic acid** exhibits antioxidant, anti-viral, anti-cancerous, and anti-inflammatory activities. Caffeic acid is commonly synthesized by a cytochrome P450 enzyme *p-*coumarate 3-hydroxylase (C3H), which catalyzes the hydroxylation of *p-*coumaric acid (Figure 3). The overexpression of *E. coli* 4-hydroxyphenylacetate 3-hydroxylase gene *hpaBC* and *Rhodobacter capsulatus* tyrosine ammonia lyase gene *tal* in a tyrosine-overproducing *E. coli* strain expressing the P_LlacO1_-controlled *tyrA*^*fbr*^*-ppsA-tktA-aroG*^*fbr*^ resulted in the production of 50.2 mg/L of caffeic acid (Lin and Yan, 2012). The titer was further enhanced to 766.68 mg/L by increasing the availability of tyrosine (Huang et al., 2013). Inclusion of the coumaroyl-CoA/caffeic -CoA biosynthesis route in the tyrosine ammonia lyase-4-coumarate 3-hydroxylase pathway did not improve caffeic acid production (Zhang and Stephanopoulos, 2013). The overexpression of *Rhodotorula glutinis tal* and *S. espanaensis* 4-coumarate 3-hydroxylase gene in a tyrosine-overproducing *E. coli* strain yielded 106 mg/L of caffeic acid in a 2-L bioreactor batch fermentation process (Zhang and Stephanopoulos, 2013). HpaB from *Pseudomonas aeruginosa* and HpaC from *Salmonella enterica* along with Tal from *Rhodosporidium toruloides* was overexpressed in *S. cerevisiae* for the production of caffeic acid, which reached 289.4 mg/L (Liu L. et al., 2019). This is the first report on engineering *S. cerevisiae* for the production of caffeic acid. The highest level of caffeic acid (10.2 g/L) could be produced from *p*-coumaric acid by using whole-cell catalysis of engineered *E. coli* (Furuya and Kino, 2014). This level was much higher than that obtained from glucose, indicating that the availability of *p*-coumaric acid and tyrosine might be key factors influencing caffeic acid production in *E. coli*. From the caffeic acid yield reported in the literature, the titer using whole-cell catalysis is much higher than that obtained by using fermentation. This reason may be the high toxicity of caffeic acid on host strain.

***p*-Hydroxystyrene** is a derivative of styrene. It has the same applications as styrene. The *de novo* biosynthesis of *p*-hydroxystyrene from glucose was achieved in *E. coli* by the decarboxylation of p-coumaric acid (Figure 3). The L-Phe overproducing strain *E. coli* ATCC 31884 expressing *R. glutinis pal* and the *p*-coumaric acid decarboxylase gene *pdc* from *L. plantarum* produced 0.40 g/L of *p*-hydroxystyrene in a 14-L fermenter (Qi et al., 2007). Recently, an artificial biosynthetic pathway was established in *E. coli* to produce *p*-hydroxystyrene using the tyrosine ammonia lyase gene *tal* from *S. espanaensis* and phenolic acid decarboxylase gene *pad* from *Bacillus amyloliquefaciens* (Kang et al., 2015). The overexpression of this pathway in the L-Tyr producing *E. coli* strain (chromosome-expressing *aroG*^*fbr*^ and *tyrA*^*fbr*^ in the *tyrA* deletion strain) resulted in the production of 355 mg/L of *p*-hydroxystyrene in shake flask cultures after 36 h. The introduction of the *tal*, 4-coumarate 3-hydroxylase gene *sam5* from *S. espanaensis* and *pad* genes or the *tal, sam5*, caffeic acid methyltransferase gene *com* from *A. thaliana* and *pad* genes into the above L-Tyr producing *E. coli* strain led to the production of 63 mg/L of **3,4-dihydroxystyrene** or 64 mg/L of **4-hydroxy-3-methoxystyrene** in shake flask cultures after 36 h, respectively (Kang et al., 2015). Since phenolic acid decarboxylase genes are widely distributed in various *Streptomyces* species, *Streptomyces* strains have been successfully used as the hosts for *p*-hydroxystyrene production. After comparing the phenolic acid decarboxylase gene *pad* obtained from *S. sviceus, S. hygroscopicus*, and *S. cattleya, pad* from *S. sviceus* and *tal* from *R. sphaeroides* were used to create the biosynthetic pathway of *p*-hydroxystyrene (Noda et al., 2015). The introduction of this pathway into the endoglucanase-screening *S. lividans* species directly yielded 250 mg/L *p*-hydroxystyrene from cellulose. This research group also engineered *S. mobaraense* for *p*-hydroxystyrene production, which yielded 273 mg/L of the product from glucose (Fujiwara et al., 2016a). The titer was obtained by introducing a single gene that encodes Tal from *R. sphaeroides*. *P. putida* S12 has also been used as the host strain for *p*-hydroxystyrene production. The overexpression of the bifunctional enzyme gene *pal/tal* from *R. toruloides* and *pdc* from *L. plantarum* in the *pcs* knockout strain of *P. putida* S12 resulted in the production of 147 mM (17.6 g/L) of *p*-hydroxystyrene in a two-phase fed-batch fermentation process (Verhoef et al., 2009).

In plants, hydroxycinnamic acids are commonly conjugated with quinic acid, shikimic acid, malic acid, and glycerol (Kim B. G. et al., 2013). Hydroxycinnamic acid conjugates have stronger anti-oxidative activity than hydroxycinnamic acid. Hydroxycinnamic acid conjugates are synthesized by hydroxycinnamoyl transferase (Figure 3). It has been demonstrated that the activity of hydroxycinnamate-CoA shikimate transferase (HST) from *Nicotiana tabacum* to *p*-coumaroyl-CoA is higher than that to other acyl donors (such as caffeoyl-CoA and feruloyl-CoA) (Kim B. G. et al., 2013). They also found that that activity of hydroxycinnamate-CoA quinate transferase (HQT) from *N. tabacum* to caffeoyl-CoA is higher than that to other acyl donors (such as coumaroyl-CoA and feruloyl-CoA). This group constructed an *E. coli* strain overexpressing HQT from *N. tabacum* and 4CL from *Oryza sativa* for the production of **chlorogenic acid** from caffeic acid for the first time (Figure 3). The recombinant *E. coli* strain produced 450 mg/L chlorogenic acid from caffeic acid (Kim B. G. et al., 2013). Then, this group constructed an *E. coli-E. coli* culture system. The chlorogenic acid producing *E. coli* strain was co-cultured with a caffeic acid producing *E. coli* strain for the *de novo* production of chlorogenic acid, which reached 78 mg/L (Cha et al., 2014). They also engineered an *E. coli* strain for the *de novo* synthesis of *p-*coumaroyl shikimate, which reached 236 mg/L.

**Curcumin** is a nature phenylpropanoid from the plant *Curcuma longa*. It has diverse therapeutic properties including anti-oxidant, anti-cancer, anti-inflammatory, anti-Alzeimer's, Anti-HIV, anti-Parkinson, and cholesterol-lowering activities. Rodrigues's group constructed an engineered *E. coli* expressing *A. thaliana 4CL, C. longa* diketide-CoA synthase gene (*DCS*) and *C. longa* curcumin synthase gene (*CURS1*) for the production of curcumin from ferulic acid (Figure 3) (Rodrigues et al., 2015). In this study, they also constructed an artificial biosynthetic pathway from L-tyrosine via caffeic acid by caffeoyl-CoA 3-O-methyl-transferase (CCoAOMT) from *Medicago sativo*. Curcumin can also be produced from caffeic acid, *p*-coumaric acid or tyrosine, respectively. Then, after the optimization of culture conditions, the resulting strain produced 959.3 mM (353 mg/L) curcumin from ferulic acid in shake flask cultures (Couto et al., 2017). Recently, it was demonstrated that the curcumin titer obtained through the ferulic acid pathway by caffeic acid O-methyltransferase (COMT) was higher than that obtained through the caffeic acid by CCoAOMT (Rodrigues et al., 2020). In this study, they established a two-strain coculture system to further improve the production of curcumin from L-Tyr, which reached 43.2 mM (15.9 mg/L). This titer is the highest titer of curcumin obtained from L-Tyr to date. The coculture increased the production of curcumin by 6.6-fold compared to the mono-culture. An artificial curcumin biosynthetic pathway was integrated into the chromosome of the engineered L-Tyr overproducing *E. col*i ΔCOS1 strain for the *de novo* production of curcumin from glucose (Kang et al., 2018). Then, multiplex automated genome engineering (MAGE) was applied for balancing the expression of the six enzymes of the biosynthetic pathway. The resulting strain produced 3.8 mg/L curcumin from glucose in shake flask cultures.

**L-DOPA** is an aromatic compound derived from L-tyrosine (Figure 3). L-DOPA has been used to treat Parkinson's disease, which is caused by deficiency of the neurotransmitter dopamine. Nakagawa et al. (2011) constructed an *E. coli* expressing the *Streptomyces castaneoglobisporus* tyrosinase gene *tyr* and native *tyrA*^*fb*^*, aroG*^*fbr*^*, tktA*, and *ppsA*, which could produce 293 mg/L of L-DOPA from glucose. The authors also co-expressed *tyr* from *S. castaneoglobisporus* and L-DOPA decarboxylase gene *dodc* from *P. putida* KT2440 in the L-Tyr producing *E. coli* strain, resulting in the production of 260 mg/L of **dopamine**. Munoz et al. (2011) reported an engineered *E. coli* with HpaBC activity, which could produce 1.5 g/L of L-DOPA from glucose. This titer was achieved by the overexpression of *E. coli* W *hpaBC*, native *tktA, atoG*^*fbr*^*, pheA*, and *Zymomonas mobilis tyrC* in the phosphotransferase system and *tyrR* knockout strain. Our group first used the singleplex genome engineering approach to create an L-DOPA-producing strain, *E. coli* DOPA-1 (Wei et al., 2016). MAGE based on 23 targets was then used to further improve L-DOPA production. The resulting strain, *E. coli* DOPA-30N, produced 8.67 g/L of L-DOPA within 60 h in a 5 L fed-batch fermentation process (Wei et al., 2016). Overexpression of *E. coli* W *hpaBC* along with the enhancement of the expression level of the downstream tyrosine biosynthetic pathway of shikimate in the *tyrR/pheA/serA* knockout *E. coli* strain resulted in producing 12.5 g/L L-DOPA in a 5-L bioreactor fed-batch fermentation (Das et al., 2018). To increase the catalytic efficiency of HpaBC, HpaB from *E. coli* was directly evolved and three mutants with higher activity were obtained (Fordjour et al., 2019). Overexpression of the mutant *hpaB*^*G*295*R*^*C* in an L-Tyr producing *E. coli* strain produced 25.53 g/L L-DOPA in a 5-L bioreactor fed-batch fermentation.

### Tryptophan Derivatives

**Figure 4:**
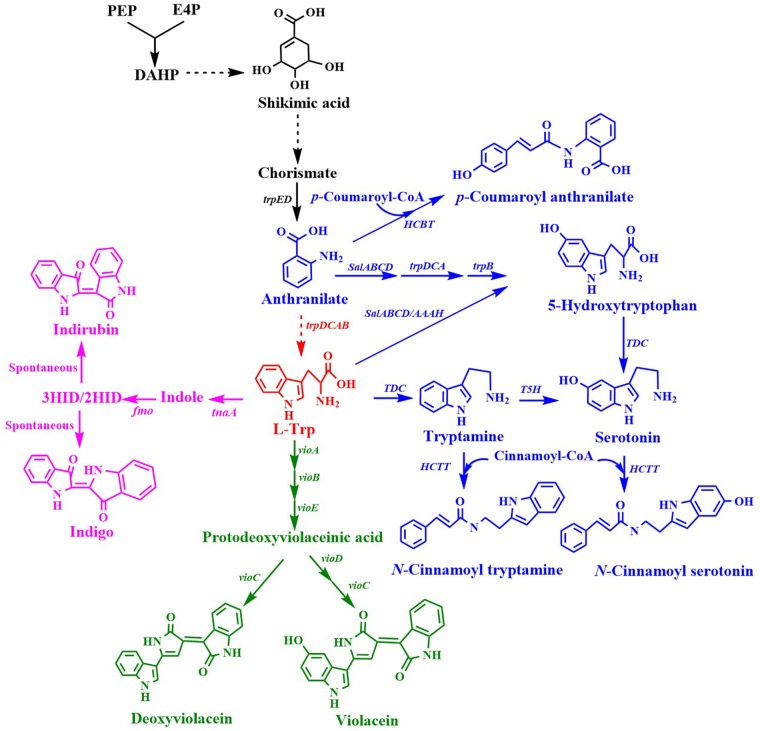
Biosynthesis of L-tryptophan derivatives. *HCBT*, anthranilate N-hydroxycinnamoyl/benzoyltransferase gene; *salABCD*, salicylate 5-hydroxylase gene; *AAAH*, aromatic amino acid hydroxylase gene; *TDC*, tryptophan decarboxylase gene; *T5H*, tryptamine 5-hydroxylase gene; *tnaA*, tryptophanase gene; *fmo*, flavin-containing monooxygenase gene; *HCTT*, hydroxycinnamoyl-CoA:tryptamine N-hydroxycinnamoyl transferase gene; *vioA*, tryptophan 2-monooxygenase/L-tryptophan oxidase gene*; vioB*, 2-imino-3-(indol-3-yl)propanoate dimerase gene*; vioE*, violacein biosynthesis protein gene; *vioC*, violacein synthase gene; *vioD*, protodeoxyviolaceinate monooxygenase gene.

L-Trp is a common precursor for the production of many important aromatic chemicals (Figure 4). A few L-Trp derivatives that have been synthesized by engineering microorganisms are listed in Table 3.

**Table 3:** *De novo* production of tryptophan derivatives by engineered microorganisms.

**Product**	**Host**	**Application**	**Strategies**	**Culture conditions**	**Titer**	**Yield**	**References**
Anthranilic acid	*E. coli* W3110 trpD9923	Synthesizing aromatic compounds	Expression *aroG^*fbr*^* and *tktA* on a plasmid	Fed-batch fermentation	14 g/L		Balderas-Hernandez et al., 2009
	*P. putida*		Δ*trpDC*ΔpheA Expressing *aroG^*D*146*N*^* and *trpE^*S*40*F*^G*	Fed-batch fermentation	1.54 g/L		Kuepper et al., 2015
Serotonin	*E. coli*	Treating various diseases related to serotonin imbalance	Expressing *Catharanthus roseus* tryptophan decarboxylase gene (*TDC*) and *O. sativa* tryptamine 5-hydroxylase gene (*T5H*)		24 mg/L		Park et al., 2011
		*E. coli*	5-hydroxytryptophan producing strain: L-Trp producing strain S028, Δ*trpR*, expressing pterin-4 alpha-carbinolamine dehydratase (PCD) and dihydropteridine reductase (DHPR) from human; expressing aromatic amino acid hydroxylase mutant (AAAH^F197L/E219C^) from *Cupriavidus taiwanensis* Serotonin producing strain: Δ*tnaA*, expressing *C. roseus TDC*	1.5 L bioreactor stepwise fermentation	154.3 mg/L		Mora-Villalobos and Zeng, 2018
N-Cinnamoyl tryptamine	*E. coli* BL 21(DE3)	Antioxidant, anti-inflammatory, anti-atherogenic and skin-whitening agents	Δ*tyrR* Expressing *PAL* from *A. thaliana* on a PACYCDuet-1 plasmid. Expressing *4CL* from *O. sativa* and hydroxycinnamoyl-CoA:tryptamine N-hydroxycinnamoyl transferase gene (*HCTT*) from *Capsicum annuumon* on a pCDFDuet-1 plasmid. Expressing tryptophan decarboxylase gene (*tdc*) from *Bacillus atrophaeus* on a pGEX5X-3 plasmid	Shake-flask fermentation	110.6 mg/L		Lee et al., 2017
*p-*coumaroyl anthranilate	*E. coli* BL 21(DE3)	Antioxidant, anti-inflammatory, anti-atherogenic, and skin-whitening agents	Δ*tyrR, ΔtrpD* Expressing 4CL from *O. sativa* and anthranilate N-hydroxycinnamoyl/benzoyltransferase (HCBT) from *Dianthus caryophyllus* on a pCDFDuet-1 plasmid. Expressing aroG, *S. espanaensis tal* and tyrA on a pACYCDuet-1 plasmid. Expressing trpEG on a pETDuet-1 plasmid	Shake-flask fermentation	317.2 mg/L		Lee et al., 2018
5-Hydroxytryptophan	*E. coli* S028 (L-Trp producing strain)	Synthesizing serotonin	Δ*trpR*, expressing PCD and DHPR from human; expressing AAAH^F197L/E219C^ from *C. taiwanensis*	1.5 L bioreactor fed-batch fermentation	962 mg/L		Mora-Villalobos and Zeng, 2018
	*E. coli* BL 21(DE3)		Δ*tnaA* Expressing *mtrA, PTPS*, and *SPR* from human, *PCD-DHPR*, and the N145 truncated mutant *TPH1* from human on a p15A origin plasmid; Expressing *aroH^*fbr*^-trpE^*fbr*^DCBA* on a pSC101 origin plasmid	10 L bioreactor fed-batch fermentation	5.1 g/L		Wang et al., 2018
Indirubin	*E. coli*	Drugs for treating chronic granulocytic leukemia, cancer, and Alzheimer's disease	Δ*trpR, ΔpykFA, P_*tktA*_::P_*trc*_*. Expressing the *trc* promoter-controlled *Methylophaga aminisulfidivorans* flavin-containing monooxygenase gene *fmo* and the *trc* promoter-controlled *E. coli* tryptophanase gene *tnaA* on a pTrc99A plasmid. Expressing the *tac* promoter-controlled *aroG^*fbr*^*, the *tac* promoter-controlled *trpE^*fbr*^* and the *tac* promoter-controlled *aroL* on a p15A origin plasmid	Fed-batch fermentation	56 mg/L		Du et al., 2018
Indigo	*E. coli*	Dye	Δ*trpR, ΔpykFA, P_*tktA*_::P_*trc*_*. Expressing the *trc* promoter-controlled *M. aminisulfidivorans* flavin-containing monooxygenase gene *fmo* and the *trc* promoter-controlled *E. coli* tryptophanase gene *tnaA* on a pTrc99A plasmid. Expressing the *tac* promoter-controlled *aroG^*fbr*^*, the *tac* promoter-controlled *trpE^*fbr*^* and the *tac* promoter-controlled *aroL* on a p15A origin plasmid	Fed-batch fermentation	640 mg/L		Du et al., 2018
Violacein	*E. coli*	Antioxidant, anti-microbial, anticancer, antiviral, trypanocidal, antiprotozoan agents	Δ*trpR, ΔtnaA, ΔsdaA, Δlac::P_*tac*_aroFBL, ΔtrpL::trpE^*fbr*^, Δgal::P_*tac*_tktA, Δxyl::P_*tac*_serA^*fbr*^, Δfuc::P_*tac*_vioD* Expressing *vioABCE* from *Chromobacterium violaceum* on a P_BAD_ plasmid	1 L bioreactor fed-batch fermentatio	710 mg/L		Rodrigues et al., 2013
			Δ*trpR* Expressing the *vioABCDE* gene cluster from *Duganella* sp. B2 on a pRSFDuet plasmid Expressing trpE^fbr^-trpD on pACYCDuet plasmid	5 L bioreactor batch fermentation	1.75 g/L	0.116 g/g	Fang et al., 2015
			Δ*trpR, ΔtnaA, ΔpheA* Expressing the *vioABCE* gene cluster and *vioE* from *Duganella* sp. B2 on a pRSFDuet plasmid Expressing *trpE^*fbr*^-trpD-serA^*fbr*^-aroG^*fbr*^* on pACYCDuet plasmid	5 L bioreactor fed-batch fermentation	4.45 g/L		Zhou et al., 2018
	*E. coli* BL21(DE3)		Plackett-Burman and Box-Behnken design was used to optimize the expression of the vioA, vioB, vioC, vioD, and vioE from *C.violaceum* 12472	2 L bioreactor fed- batch fermentation	1.31 g/L		Xu et al., 2017
	*E. coli BL21 (DE3)*		Expressing *C.violaceum vioABDE* on a ColE1 origin plasmid, *Janthinobacterium lividum vioC* on a p15A origin plasmid Knocking down *ytfR* using synthetic sRNA	1.2 L bioreactor fed-batch fermentation	5.19 g/L		Yang et al., 2019
	*Corynebacterium glutamicum* ATCC 21850 (L-Trp producing strain)		Expressing *vio* operon from *C. violaceum*	3 L bioreactor fed-batch fermentation	5.436 g/L		Sun et al., 2016
Deoxyviolacein	*E. coli*	Antioxidant, anti-microbial, anticancer, antiviral, trypanocidal, antiprotozoan agents	Δ*trpR, ΔtnaA, ΔsdaA, Δlac::P_*tac*_aroFBL, ΔaraBAD, ΔtrpL::trpE^*fbr*^, Δgal::P_*tac*_tktA, Δxyl::P_*tac*_serA^*fbr*^* Expressing vioABCE from *C. violaceum* on a P_BAD_ plasmid	1 L bioreactor batch fermentatio	1.6 g/L		Rodrigues et al., 2014
	*E. coli* BL21 (DE3)		Δ*trpR, ΔtnaA, ΔpheA* Expressing the *vioABCE* gene cluster from *Duganella* sp. B2 on a pRSFDuet plasmid Expressing *trpE^*fbr*^-trpD-serA^*fbr*^-aroG^*fbr*^* on pACYCDuet plasmid	5 L bioreactor fed-batch fermentation	1.92 g/L		Fang et al., 2016

**Indirubin**, also known as couraupitine B, is an indole alkaloid that is a major bioactive ingredient of a traditional oriental medicine called Danggui Longhui Wan. It is currently used as a drug to treat chronic granulocytic leukemia and may have therapeutic value in the treatment of cancer and Alzheimer's disease. Indirubin is derived from L-Trp, which is converted into indole by native tryptophanase, then reduced to 2-hydroxyindole (2HID) and 3-hydroxyindole (3HID) by heterologous monooxygenase or dioxygenase, and finally undergoes spontaneous dimerization with 3HID and 2HID to form indirubin (Figure 4). Recently, an artificial *de novo* biosynthetic pathway was developed for the production of 56 mg/L indirubin and 640 mg/L indigo from glucose (Du et al., 2018). The titer was achieved by the overexpression of *Methylophaga aminisulfidivorans* flavin-containing monooxygenase gene *fmo* and *E. coli* tryptophanase gene *tnaA* as well as by enhancing the expression of the feedback-resistant *aroG*^*fbr*^ and *trpE*^*fbr*^ in the *trpR/pykFA* knockout *E. coli* strain. This was the first report about the direct production of indirubin from glucose in a shake flask culture.

**Violacein** and **deoxyviolacein** are secondary metabolites that receives high interest due to their biological activities such as anti-microbial, anti-cancer, anti-viral, trypanocidal, anti-protozoan, and anti-oxidant activities (Fang et al., 2015). *E. coli* expressing the *vioABCE* cluster from *Chromobacterium violaceum* under control of the inducible *araC* system produced 180 mg/L deoxyviolacein from glucose (Figure 4) (Rodrigues et al., 2013). System-level analysis for the serine, chorismate and L-Trp biosynthesis and the non-oxidative pentose phosphate pathway revealed the bottlenecks in L-Trp biosynthesis. After eliminating the bottlenecks in L-Trp supply, the deoxyviolacein titer was further improved to 320 mg/L. Moreover, co-expression of the *vioD* from *C. violaceum* in the above deoxyviolacein producing strain dVio-6 led to the production of 710 mg/L violacein from glucose in a fed-batch fermentation (Rodrigues et al., 2013). In their further study (Rodrigues et al., 2014), the L-arabinose metabolism of the above deoxyviolacein producing strain dVio-6 was eliminated to prevent catabolism of the inducer L-arabinose by knocking the *araBAD* genes. The resulting *E. coli* strain dVio-8 produced 1.6 g/L deoxyviolacein from glycerol in a fed-batch fermentation (Rodrigues et al., 2014). Using another *vioABCE* cluster from *Duganella* sp. B2, an engineered *E. coli* strain was used to produce violacein (Fang et al., 2015). Co-overexpression of this gene cluster, *trpE*^*M*293*T*/*S*40*L*^ and *trpD* in the *trpR* deletion *E. coli* strain led to the production of 1.75 g/L violacein in a 5 L bioreactor batch fermentation (Fang et al., 2015). This group then developed an intermediate sensor-assisted push-pull strategy (InterSPPS) (Fang et al., 2016). In this study, an L-Trp biosensor was first developed. Then the L-Trp biosensor was used to sequentially enhance the upstream and downstream modular of L-Trp in the deoxyviolacein biosynthetic pathway. By this means, the deoxyviolacein titer was increased by 4.4-fold (1.92 g/L) (Fang et al., 2016). This group's study also demonstrated that VioE is the rate-limiting enzyme for the violacein biosynthesis (Zhou et al., 2018). Overexpression of the *vioE* further improved the production of violacein. The final recombinant *E. coli* strain produced 4.45 g/L violacein in 5-L bioreactor fed-batch fermentation (Zhou et al., 2018). A statistical model-based multivariate regulator strategy was developed to improve metabolic pathway efficiency (Xu et al., 2017). A combination of Plackett-Burman design with Box-Behnken design was used to optimize the expression levels of the violacein biosynthetic pathway genes by using different strength promoters. The final *E. coli* strain produced 1.31 g/L of violacein in a 2 L bioreactor fed-batch fermentation (Xu et al., 2017). Constraints-based flux balance analysis (FBA) identified tryptophan biosynthesis and NADPH availability as limiting (Immanuel et al., 2018). The recombinant *E. coli* strain based on FBA produced a 6-fold increase in violacein yield. The synthetic small regulatory RNA (sRNA) technology has been successfully used for genome-scale screening of beneficial knockdown gene targets in a violacein producing *E. coli* strain (Yang et al., 2019). As a result, knocking down *ytfR* (encoding sugar ABC transporter) increased 600% violacein titer. The *ytfR* knockdown strain produced 5.19 g/L violacein in fed-batch fermentation. *Corynebacterium glutamicum* has been successfully used as a host strain for the production of violacein. Introduction of the *vio* operon from *C. violaceum* into the L-Trp producer *C. glutamicum* ATCC 2185 led to the production of 5.436 g/L violacein in a 3 L bioreactor fed-batch fermentation (Sun et al., 2016). The value is the highest to date.

## Production of Stilbenes

**Figure 5:**
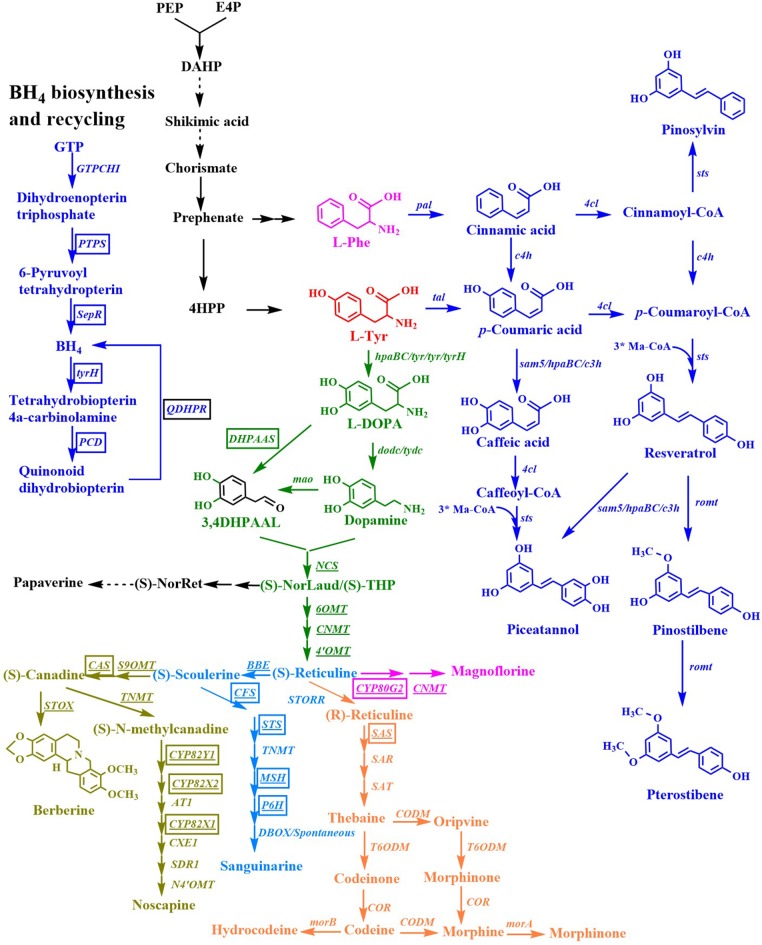
Biosynthesis of stilbenes, benzylisoquinoline alkaloids. Black, microorganism genes; Boxed genes, mammalian (*Rattus norvegicus/ Bombyx mori*) genes; Underlinded, plant genes; Boxed and underlined genes, cytochrome P450 genes; Ma-CoA, malonyl-CoA; (S)-NorLaud, (S)-norlaudanosoline; (S)-THP, (S)-tetrahydropapaveroline; (S)-NorRet, (S)-norreticuline; BH_4_, tetrahydrobiopterin; *pal*, phenylalanine ammonia lyase gene*; 4cl*, 4-coumaroyl-coenzyme A ligase gene; *sts*, stilbene synthase gene; *c4h*, 4-cinnamic acid hydroxylase gene; *sam5*, 4-coumarate hydroxylase gene; *c3h*, p-coumarate 3-hydroxylase gene; *romt*, resveratrol O-methyltransferase; *tyr/tyrH*, tyrosinase gene; *DHPAAS*, 3,4-dihydroxyphenylacetaldehyde synthase gene; *dodc*, DOPA decarboxylase gene; *tydc*, tyrosine decarboxylase gene; *mao*, monoamine oxidase gene; *NCS*, norcoclaurine synthase gene; *6OMT*, norcoclaurine 6-O-methyltransferase gene; *CNMT*, coclaurine-N-methyltransferase gene; *4'-OMT*, 3'-hydroxy-N-methylcoclaurine-4'-O-methyltransferase gene; *CYP80G2*, corytuberine synthase gene; *BBE*, berberine bridge enzyme gene; *GTPCHI*, GTP cyclohydrolase I gene; *PTPS*, 6-pyruvoyl-tetrahydropterin synthase gene; *SepR*, sepiapterin reductase gene; *PCD*, pterin-4α-carbinolamine dehydratase gene; *QDHPR*, quinonoid dihydropteridine reductase gene; *S9OMT*, scoulerine 9-O-methyltransferase gene; *CAS*, canadine synthase gene; *CPR*, cytochrome P450 reductase gene; *TNMT*, tetrahydroprotoberberine cis-N-methyltransferase gene; *CYP82Y1*, 1-hydroxy-N-methylcanadine synthase gene; CYP82X2, 1- hydroxy-N-methylcanadine 13-hydroxylase gene; *AT1*, 1,13-dihydroxy-N-methylcandine 13-O-acetyltransferase gene; CYP82X1, 1-hydroxy-13-O-acetyl-N-methylcanadine 8-hydroxylase gene; *CXE1*, 3-O-acetylpapaveroxine carboxylesterase gene; *SDR1*, short-chain dehydrogenase/reductase gene; *N4'-OMT*, narcotoline-4′-O-methyltransferase gene; *CFS*, cheilanthifoline synthase gene; *STS*, stylopine synthase gene; *TNMT*, tetrahydroprotoberberine N-methyltransferase gene; *MSH*, cis-N-methylstylopine 14-hydroxylase gene; P6H, protopine 6-hydroxylase gene; *DBOX*, dihydrobenzophenanthridine oxidase gene; *STOX*, (S)-tetrahydroprotoberberine oxidase gene; *STORR*, (S)-reticuline isomerase gene; *SAS*, salutaridine synthase gene; *SAR*, salutaridinol reductase gene; *SAT*, salutaridinol acetyltransferase gene; *CODM*, codeine-O-demethylase gene; *T6OMT*, thebaine 6-O-demethylase gene; *COR*, codeinone reductase gene; *morA*, morphine dehydrogenase; *morB*, morphine reductase.

Stilbenes are composed of a skeleton with two aromatic rings joined by a methylene bridge. They are biosynthesized through the condensation of Co-A-linked cinnamic acids with three molecules of malonyl-CoA catalyzed by stilbene synthase (Figure 5, Table 4). Pinosylvin, resveratrol, and piceatannol are the three stilbenoids derived from cinnamic acid, *p*-coumaric acid, and caffeic acid, respectively. Recently, some 4-coumaroyl-coenzyme A ligases (4CLs) and stilbene synthase (STS) were introduced into microorganisms for the production of resveratrol, pterostilbene, piceatannol, and pinosylvin (Shrestha et al., 2019; Thapa et al., 2019). In most studies, **resveratrol** was produced from *p*-coumaric acid by the co-expression of 4CL and STS (Beekwilder et al., 2006; Watts et al., 2006; Katsuyama et al., 2007b; Lim et al., 2011), or from L-Tyr (or L-Phe) by the co-expression of Tal (or Pal), 4CL, and STS (Katsuyama et al., 2007a; Wu et al., 2013; Wang S. Y. et al., 2015). The highest titer (2.3 g/L) of resveratrol was obtained from *p*-coumaric acid by engineered *E. coli* BW27784 containing the *4cl1* gene from *A. thaliana* and *sts* gene from *Vitis vinifera* in the presence of 0.05 mM cerulenin (Lim et al., 2011). The authors also demonstrated that the combination of 4CL from *A. thaliana* and STS from *V. vinifera* was the most suitable combination for resveratrol production. The *de novo* biosynthesis of resveratrol from glucose was reported for the first time by Liu et al. (2016). The *tal* gene from *R. glutinis, 4cl* gene from *P. crispum*, and *sts* gene from *V. vinifera* were site-specifically integrated into the loci of the genes *tyrR* and *trpED* within the chromosome of *E. coli* BW25113 (DE3). The engineered strain produced 4.612 mg/L of resveratrol from glucose in shake flask cultures (Liu et al., 2016). A co-culture of *p-*coumaric acid producing *E. coli* and resveratrol producing *E. coli* was used to improve resveratrol production from glycerol to 22.6 mg/L in a 1-L bioreactor batch fermentation process (Camacho-Zaragoza et al., 2016). Recently, the CRISPRi system was used to repress *fabFBID* involved in the fatty acid biosynthetic pathway, which improved the amount of resveratrol produced from glucose to 304.5 mg/L (Wu et al., 2017b). The titer was obtained by the introduction of the malonate assimilation pathway from *Rhizobium trifolii* and the repression of the fatty acid biosynthetic pathway using CRISPRi. Since 2003, *S. cerevisiae* has been successfully used as the host strain for stilbene production from *p-*coumaric acid, by the introduction of the 4*CL216* gene from a hybrid poplar and the *vst1* gene from *V. vinifera* (Becker et al., 2003). Li et al. (2015) first reported an engineered *S. cerevisiae* capable of the *de novo* biosynthesis of resveratrol from glucose. The introduction of the *tal* gene from *Herpetosiphon aurantiacus, 4cl* gene from *A. thaliana*, and *sts* gene from *V. vinifera* in an engineered *S. cerevisiae* overexpressing the feedback-insensitive alleles of *ARO4* and *ARO7* resulted in the *de novo* production of resveratrol from glucose (Li et al., 2015). The overexpression of the inactivation-resistant variant of acetyl-CoA carboxylase (Acc1p^S659A/S1157A^) to abolish its phosphorylation, and the increase in the copy number of the heterologous resveratrol biosynthetic pathway via tyrosine further improved the amount of resveratrol produced from glucose to 415.65 mg/L, or that produced from ethanol to 532.42 mg/L, in a fed-batch fermentation process. The authors then applied a strategy involving pull-push-block stain engineering to further improve the amount of resveratrol produced from glucose in a fed-batch fermentation process to 812 mg/L (Li M. et al., 2016). The titer was obtained by the multiple integrations of the heterologous pathway genes via phenylalanine, enhancement of P450 activity, upregulation of the expression levels of the feedback-insensitive alleles of *ARO4* and *ARO7*, and the inactivation-resistant variant of acetyl-CoA carboxylase (Acc1p^S659A/S1157A^), along with blockage of the decarboxylation of phenylpyruvate. Kallscheuer et al. (2016) constructed a *C. glutamicum* platform strain for the production of resveratrol from glucose. The heterologous biosynthetic pathway genes containing the *sts* gene from *Arachis hypogaea, 4cl* gene from *P. crispum* and *tal* gene from *Flavobacterium johnsoniae*, and the *aroH* gene from *E. coli* were introduced into the four gene cluster knockout *C. glutamicum* strain. The final strain produced 59 mg/L of resveratrol from glucose in the presence of 25 μM of cerulenin in shake flask cultures (Kallscheuer et al., 2016). Then, this group found that increasing the intracellular acetyl-CoA availability through the reduction of citrate synthase activity further improved the production of resveratrol to 112 mg/L (Milke et al., 2019). A RppA-coupled malonyl-CoA biosensor was developed for high-throughput screening of targets increasing the malonyl-CoA pool (Yang et al., 2018). The authors applied the biosensor to screen an 1858 synthetic sRNA library and found 14 knockdown targets that generally enhanced malonyl-CoA level in *E. coli*. The *pabA* knockdown using sRNA increased resveratrol production by 4.2-fold, which achieved 51.8 mg/L (Yang et al., 2018). After screening pathway genes from various species and exploring their expression pattern, an artificial resveratrol biosynthetic pathway was created and was introduced into *E. coli* strain for the production of resveratrol (Zhao et al., 2018). Co-overexpression of chaperone protein genes (*groeS-groEL*), transport gene (*ompF*), and malony-CoA biosynthetic pathway genes (*accBC-dtsR1*) from *C. glutamicum* along with antisense inhibiting *fabD* significantly increased the production of resveratrol from L-Tyr, which reached 238.71 mg/L.

**Table 4:** Production of stilbenes and benzylisoquinoline alkaloids by engineered microorganism.

**Products**	**Host**	**Genes expressed/deleted**	**Precursor**	**Culture conditions**	**Titer (mg/L)**	**References**
**Stilbenes**						
Resveratrol	*E. coli* BW27784	Expressing *Arabidopsis thaliana 4cl* and *Vitis vinifera sts* on a pUC18 plasmid	*p*-coumaric acid	Shake-flask fermentation in the presence of 0.05 mM cerulenin	2.3 g/L	Lim et al., 2011
Resveratrol	*E. coli* BW25113 (DE3)	Δ*tyrR::P_*T*7_-sts, ΔtrpED::P_*T*7_-tal- P_*T*7_-4cl*	Glucose	Shake-flask fermentation	4.612 mg/L	Liu et al., 2016
Resveratrol	*E. coli-E. coli*	The *pheA* knockout strain of W3110 expressing the *aroG^*fbr*^* and *tktA* on a pACYC184 origin plasmid, and the *pal* gene from *R. glutinis* on a pTrc99A plasmid; Another W3110 strain expressing the *4CL* gene from *S. coelicolor* A2 and the *STS* gene from *A. hypogaea* on a pTrc99A plasmid	Glycerol	1L bioreactor batch coculture fermentation	22.6 mg/L	Camacho-Zaragoza et al., 2016
Resveratrol	*E. coli* BL21 (DE3)	*ΔlacZ::P_*T*7_aroG^*fbr*^-tyrA^*fbr*^*. Expressing *tal* from *Trichosporon cutaneum* and *4cl* from *P. crispum* on a CloDF13 origin plasmid. Expressing *sts* from *V. vinifera* on a pET22b plasmid. Expressing *matB* and *matC* from *Rhizobium trifolii* on a pACYCDuet-1 plasmid. Repressing *fabF, fabI, fabB*, and *fabD* using CRISPRi	Glucose	Shake-flask fermentation	304.5 mg/L	Wu et al., 2017b
	*E. coli* BL21 (DE3)	Expressing *TAL* from *Phanerochaete chrysosporium* and *4CL* from *A. thaliana* on pRSFDuet-1 plasmid. Expressing *V. vinifera STS* on pCDFDuet-1 plasmid. Expressing *groES-groEL* on pACYC origin plasmid. Expressing *C. glutamicum accBC-dtsR1*, P_T7_ anti-*fabD*, and *ompF* on pETDuet-1 plasmid	L-Tyr	Shake-flask fermentation	238.71 mg/L	Zhao et al., 2018
Resveratrol	*S. cerevisiae*	Chromosomal expressing P_TEF1_-*ARO7^*G*141*S*^, P_*PGK*1_-ARO4^*K*229*L*^*, and *P_*TEF*1_-ACC1^*S*659*A*/*S*1157*A*^*, multiple-integration of the heterologous pathway genes consisted of the *tal* gene from *Herpetosiphon aurantiacus*, the *4cl* gene from *A. thaliana* and the *sts* gene from *V. vinifera*	Glucose	1-L bioreactor fed-batch fermentation	415.65 mg/L	Li et al., 2015
Resveratrol	*S. cerevisiae*	*ΔARO10*. Chromosomal expressing P_TEF1_-*ARO7^*G*141*S*^, P_*PGK*1_-ARO4^*K*229^, P_*TEF*1_-ACC1^*S*659*A*/*S*1157*A*^*, P_TDH3_-*SeACS^*L*641*P*^, P_*TEF*1_-AtATR2*, and *P_*PGK*1_-CYB5*, multiple-integration of the heterologous pathway genes consisted of the *pal* gene from *A. thaliana*, the *c4h* gene from *A. thaliana*, the *4cl2* gene from *A. thaliana*, and the *sts* gene from *V. vinifera*	Glucose	Fed-batch fermentation	812 mg/L	Li M. et al., 2016
Resveratrol	*C. glutamicum* ATCC 13032	*Δcg0344-47, Δcg2625-40, Δcg1226, Δcg0502*.Expressing stilbene syntase gene from *Arachis hypogaea* and 4-coumarate CoA ligase gene from *P. crispum* on a pMKEx2 plasmid. Expressing *aroH* from *E. coli* and tyrosine ammonia lyase gene from *Flavobacterium johnsoniae* on a pEKEx3 plasmid	Glucose	Shake-flask fermentation with cerulenin supplementation	59 mg/L	Kallscheuer et al., 2016
	*C. glutamicum* ATCC 13032	*Δcg0344-47, Δcg2625-40, Δcg1226, Δcg0502*, Δcg0344-47:PT7-Pc4clExpressing *A. hypogaea sts* and *P. crispum 4cl* on a plasmid. Expressing *E. coli aroH* and *F. johnsoniae tal* on another plasmidReplacing the native *gltA* promotor with the *dapA* promotor variant C7	Glucose	2 L bioreactor batch fermentation	112 mg/L	Milke et al., 2019
Pinostilbene	*S. cerevisiae*	*ΔARO10*Chromosomal expressing P_TEF1_-*ARO7^*G*141*S*^, P_*PGK*1_-ARO4^*K*229^, P_*TEF*1_-ACC1^*S*659*A*/*S*1157*A*^*, P_TDH3_-*SeACS^*L*641*P*^, P_*TEF*1_-AtATR2, P_*PGK*1_-CYB5*, and *P_*TDH*3_-sbROMT*, multiple-integration of the heterologous pathway genes consisted of the *pal* gene from *A. thaliana*, the *c4h* gene from *A. thaliana*, the *4cl2* gene from *A. thaliana*, and the *sts* gene from *V. vinifera*	Glucose	Feed-in-time medium in m2p microbioreactor	5.52 mg/L	Li M. et al., 2016
Pterostilbene	*S. cerevisiae*	*ΔARO10*.Chromosomal expressing P_TEF1_-*ARO7^*G*141*S*^, P_*PGK*1_-ARO4^*K*229^, P_*TEF*1_-ACC1^*S*659*A*/*S*1157*A*^*, P_TDH3_-*SeACS^*L*641*P*^, P_*TEF*1_-AtATR2, P_*PGK*1_-CYB5*, and *P_*TDH*3_-VvROMT*, multiple-integration of the heterologous pathway genes consisted of the *pal* gene from *A. thaliana*, the *c4h* gene from *A. thaliana*, the *4cl2* gene from *A. thaliana*, and the *sts* gene from *V. vinifera*	Glucose	Feed-in-time medium in m2p microbioreactor	34.93 mg/L	Li M. et al., 2016
Pterostilbene	*E. coli* C41(DE3)	Δ*tyrR::tyrA^*fbr*^-aroG^*fbr*^*.Expressing the codon-optimized tyrosine ammonia lyase gene from *S. espanaensis, p*-coumarate:CoA ligase gene from *N. tabacum*, caffeic acid O-methyltransferase gene from *A. thaliana*, and stilbene synthase gene from *V. vinifera* on a pET-28a(+) vector	Glucose	Shake-flask fermentation with L-methionine supplementation	33.6 mg/L	Heo et al., 2017
Pinosylvin	*E. coli*	Expressing *Pinus strobus* stilbene synthase T248A mutant with an N-terminal His_6_ tag, the 4-coumarate:CoA ligase A294G mutant from *S. coelicolor* and the phenylalanine ammonialyase gene from *P. crispum* on a RSR origin plasmid (pRSFDuet1)	Glucose	Shake-flask fermentation with the addition of cerulenin and L-Phe	91 mg/L	van Summeren-Wesenhagen and Marienhagen, 2015
Pinosylvin	*E. coli* ATCC31884- *E. coli* DH5α	L-Phe producer expressing *R. glutini pal* on a pBR322 origin plasmid (pQE30). *E. coli* DH5α expressing *A. thaliana 4cl, V. vinifera rs* on a pBR322 origin plasmid (pQE30). The repression of *fabD* using CRISPRi	Glycerol		47.5 mg/L	Liang et al., 2016
Pinosylvin	*E. coli* BL21 (DE)	Expressing the T7 promoter-controlled *aroF^*wt*^*, the T7 promoter-controlled *pheA^*fbr*^* and the T7 promoter-controlled *pal* gene from *Trichosporon cutaneum* on a p15A origin plasmid (pACYCDuet-1). Expressing the T7 promoter-controlled *4cl* gene from *P. crispum* and the T7 promoter-controlled *sts* from *V. vinifera* on a pBR322 origin plasmid (pETDuet-1)	glucose	Shake-flask fermentation	281 mg/L	Wu et al., 2017a
Piceatannol	*E. coli*	Expressing *E. coli* W *hpaBC* on a pZE12-luc plasmid	Resveratrol	Shake-flask fermentation	1.2 g/L	Lin and Yan, 2014
**Benzylisoquinoline**						
(R,S)-Reticuline	*E. coli*	Expressing the T7 promoter-controlled *Coptis japonica* norcoclaurine synthase gene (*NCS*) and the *tac* promoter-controlled *M. luteus* monoamine oxidase gene (*mao*) on a pKK223-3 plasmid (pBR322 *ori*). Expressing the T7 promoter-controlled *C. japonica* norcoclaurine 6-O-methyltransferase gene (*6OMT*), the T7 promoter-controlled *C. japonica* 3'-hydroxy-N-methylcoclaurine-4'-O-methyltransferase gene (*4'OMT*) and the T7 promoter-controlled *C. japonica* coclaurine-N-methyltransferase gene (*CNMT*) on a pACYC184 plasmid	Dopamine	Shake-flask fermentation	11 mg/L	Minami et al., 2008
Magnoflorine	*E. coli- S. cerevisiae*	*E. coli*: Expressing the T7 promoter-controlled *C. japonica NCS* and the *tac* promoter-controlled *M. luteus mao* on a pKK223-3 plasmid (pBR322 *ori*). Expressing the T7 promoter-controlled *C. japonica 6OMT*, the T7 promoter-controlled *C. japonica 4'OMT* and the T7 promoter-controlled *C. japonica CNMT* on a pACYC184 plasmid.*S. cerevisiae*: Expressing *CYP80G2* and *CNMT* from *C. japonica* on a pGYR plasmid.	Dopamine	Shake-flask *E. coli- S. cerevisiae* culture	7.2 mg/L	Minami et al., 2008
Scoulerine	*E. coli- S. cerevisiae*	*E. coli*: Expressing the T7 promoter-controlled *C. japonica NCS* and the tac promoter-controlled *M. luteus mao* on a pKK223-3 plasmid (pBR322 *ori*). Expressing the T7 promoter-controlled *C. japonica 6OMT*, the T7 promoter-controlled *C. japonica 4'OMT* and the T7 promoter-controlled *C. japonica CNMT* on a pACYC184 plasmid.*S. cerevisiae*: Expressing berberine bridge enzyme gene (*BBE*) from *C. japonica* on a pYES2 plasmid.	Dopamine	Shake-flask *E. coli- S. cerevisiae* culture	8.3 mg/L	Minami et al., 2008
Reticuline	*E. coli* BL21 (DE3)	Expressing the T7 promoter-controlled *C. japonica NCS* and the T7 promoter-controlled *M. luteus mao* on a pCDFPL plasmid (CloDF13 *ori*). Expressing the T7 promoter-controlled *C. japonica 6OMT*, the T7 promoter-controlled *C. japonica 4'OMT* and the T7 promoter-controlled *C. japonica CNMT* on a pACYC184 plasmid.	Dopamine	Shake-flask fermentation	54 mg/L	Kim J. S. et al., 2013
(S)-Reticuline	*E. coli* BL21 (DE3)	Δ*tyr.*, Expressing the T7 promoter-controlled *tyrA^*fbr*^*, the T7 promoter-controlled *aroG^*fbr*^*, the T7 promoter-controlled *tktA* and the T7 promoter-controlled *ppsA* on a pCOLADuet-1 plasmid. Expressing the T7 promoter-controlled *C. japonica NCS*, the T7 promoter-controlled *Ralstonia solanacearum tyr*, the T7 promoter-controlled *P. putida dodc* and the T7 promoter-controlled *M. luteus mao* on a pET-21d plasmid. Expressing the T7 promoter-controlled *C. japonica 6OMT*, the T7 promoter-controlled *C. japonica 4'OMT* and the T7 promoter-controlled *C. japonica CNMT* on a pACYC184 plasmid.	Glycerol	Fed-batch fermentation	46 mg/L	Nakagawa et al., 2011
	*E. coli* BL21 (DE3)	Δ*tyrR*Expressing *6OMT, CNMT, DODC*, and *4′OMT* in pACYC184 plasmid, *NCS* and *MAO* in pET-23a plasmid, *Bacillus subtilis* GTP cyclohydrolase gene (*mtrA*), rat 6-pyruvoyl-tetrahydropterin synthase gene (*PTPS*), rat sepiapterin reductase gene (*SPR*), and *Drosophila melanogaster* tryrosine hydroxylase (*TH2*) in pCDFPL plasmid, and *tyrA^*fbr*^, aroG^*fbr*^, tktA*, and *ppsA* in pCOLADuet-1 plasmid		Fed-batch fermentation	163.5 mg/L	Matsumura et al., 2018
(S)-Reticuline	*S. cerevisiae*	Chromosomal integration of ARO4^fbr^, *Bete vilgaris* CYP76AD1^W13L/F309L^, *P. putida dodc* and *P. somniferum NCS*; plasmid-expressing of *6OMT, CNMT* and 4'OMT from *P. somniferum*, and *Eschscholzia californica CYP80B1* on a CEN6/ARS4 plasmid.	Glucose	Shake-flask fermentation	80.6 μg/L	DeLoache et al., 2015
(S)-Reticuline	*S. cerevisiae* CEN.PK2-1D	*trp1*Δ*:: ARO4^*Q*166*K*^; zwf1Δ; TKL1:: P_*GPD*_-TKL1; fcy1*Δ*:: KlURA3-P_*GPD*_-PpDODC-T_*ADH*1_; YBR197C*Δ*:: P_*TPI*1_-RnSepR-T_*STE*2_, P_*TEF*1_-RnPTPS-T_*CYC*1_, KanMX, P_*GPD*_-RnQDHPR-T_*AHD*1_, P_*PGK*1_-RnPCD-T_*PHO*5_; YDR514C*Δ*:: P_*PYK*1_-PsCNMT-T_*MFa*1_, P_*PGK*1_-Ps6OMT-T_*PHO*5_, P_*GPD*_-EcCYP80B1-T_*ADH*1_, LEU2, P_*TEF*1_-PsCPR-T_*CYC*1_, P_*TPI*1_-Ps4OMT-T_*STE*2_*. Expressing *Rattus norvegicus tyrH^*W*166*Y*/*R*37*E*/*R*38*E*^* on a CEN6/ARS4 plasmid. Expressing *C. japonica NCS* on a CEN6/ARS4 plasmid	Glucose	Shake-flask fermentation	19.2 μg/L	Trenchard et al., 2015
Noscapine	*S. cerevisiae*	*ura3*Δ*::(P_*TPI*1_-Ps4'OMT-T_*STE*2_, P_*TEF*1_-AtATR1-T_*CYC*1_, P_*PGK*1_-Ps6OMT-T_*PHO*5_, P_*GPD*_-CjCAS-T_*ADH*1*t*_);* * trp1*Δ*::(P_*GPD*_-PsCNMT-T_*ADH*1*t*_, P_*HXT*7_-CYP82Y1A-T_*PGK*1_, P_*TEF*1_-PsS9OMT-T_*CYC*1_, P_*PGK*1_-PsBBE-T_*PHO*5_);* * leu2*Δ*::(P_*HXT*7_-CYP82X2-T_*CYC*1_-loxP-KanMX-loxP, P_*GPD*_-PsAT1-T_*ADH*1_, P_*TPI*1_-PsSDR1-T_*STE*2_, P_*PGK*1_-PsMT3-T_*PHO*5_);* * his3*Δ*::(P_*GPD*_-PsTNMT-T_*CYC*1_, P_*PGK*1_-PsMT2-T_*PHO*5_-loxP-HygroBR, P_*HXT*7_-CYP82X1-T_*PGK*1_, P_*PYK*1_-PsCXE1-T_*MFA*1_)*, Expressing *P. somniferum CYP82X2* on a CEN6/ARS4 plasmid, Expressing *P. somniferum S9oMT* on a CEN6/ARS4 plasmid.	Norlaudanosoline	500-μL 96-deep well plate fermentation	1.64 mM	Li and Smolke, 2016
Noscapine	*S. cerevisiae*	*ybr197c*Δ*::(P_*TPI*1_-yRnSpr-T_*STE*2_, P_*TEF*1_-yRnPts-T_*CYC*1_, P_*GPD*_-yRnQdpr-T_*ADH*1_, P_*PGK*1_-yRnPcbd1-T_*PHO*5_);* * ydr514c*Δ*::(P_*PYK*1_-PsCNMT-T_*MFa*1_, P_*PGK*1_-Ps6OMT-T_*PHO*5_, P_*TDH*3_- yEcNMCH-T_*ADH*1_, P_*TEF*1_-yPsCPR-T_*CYC*1_, P_*TPI*1_-yPs4′OMT-T_*STE*2_);* * ymr206w*Δ*::(P_*GPD*_-yRntyrhWR-T_*ADH*1_, P_*TPI*1_-yPpddc-T_*STE*2_, P_*TEF*1_-yRnDhfr-T_*CYC*1_, P_*PGK*1_-truncated-yCjNCS-T_*PHO*5_);* * ybl059w*Δ*::* *(ARO4^*Q*166*K*^, ARO7^*T*226*I*^, P_*TEF*1_-ARO10- T_*CYC*1_, P_*TDH*3_-TKL1-T_*ADH*1_);* * ypl250c*Δ*::(P_*GPD*_-yRntyrh^*WR*^-T_*ADH*1_, P_*TEF*1_-Ps4′OMT-T_*CYC*1_, P_*PGK*1_-truncated-yCjNCS-T_*PHO*5_);* * trp1*Δ*::(P_*HXT*7_-CYP82Y1A-T_*PGK*1_, P_*TEF*1_-PsS9OMT-T_*CYC*1_, P_*PGK*1_-PsBBE-T_*PHO*5_, P_*GPD*_-CjCAS-T_*ADH*1_);* * HIS3*Δ*::(P_*GPD*_-PsTNMT-T_*CYC*1_, P_*PGK*1_-PsMT2-T_*PHO*5_, P_*ADH*1_-CYP82X1-T_*GAP*1_, P_*PYK*1_-PsCXE1-T_*MFA*1_);* * leu2*Δ*::(P_*HXT*7_-CYP82X2-T_*CYC*1_, P_*GPD*_-PsAT1-T_*ADH*1_, P_*TPI*1_-PsSDR1-T_*STE*2_, P_*PGK*1_-PsMT3-T_*PHO*5_);* * ura1*Δ*::(P_*TEF*1_-TYR1-T_*CYC*1_, P_*PYK*1_-CYP82X2-* *T_*PYK*1_, TmTRP, P_*GPD*_-PsS9OMT-T_*ADH*1_, P_*PYK*1_* *-ALD6-T_*MFA*1_)*	Glycerol	Shake-flask fermentation	2.2 mg/L	Li X. et al., 2018
Sanguinarine	*S. cerevisiae*	*his3*Δ*::P_*TEF*1_ -Ps6OMT, leu2*Δ*::P_*TEF*1_ –PsCNMT, ura3*Δ*::P_*TEF*1_-Ps4'OMT, trp1*Δ*::P_*TEF*1_- ATR1, lys2*Δ*::P_*TEF*1_-PsBBE-, met15*Δ*::P_*GPD*_-EcCFS-, cin5*Δ*::P_*GPD*_-EcSTS-,XI-3*Δ*::P_*GPD*_-PsTNMT-loxP-LEU2-loxP, XI-4*Δ*::P_*GPD*_-PsMSH-loxPKanMX-loxP*,Expressing *E. californica F6H* on a low-copy plasmid.	(R,S)-norlaudanosoline		80 μg/L	Trenchard and Smolke, 2015
(S)-Canadine	*S. cerevisiae*	*his3*::P_TEF1_-*Ps6OMT*-T_CYC1_, *leu2*::P_TEF1_-*PsCNMT*-T_CYC1_, *ura3*::P_TEF1_-*Ps4′OMT*-T_CYC1_, *trp1*::P_TEF1_-*CPR*-T_CYC1_, *lys2*::P_TEF1_-*PsBBE*-T_CYC1_,Expressing *9OMT* from *Thalictrum flavum* on a 2 μ plasmid. Expressing *P. somniferum BBE*, the codon-optimized *P. somniferum S9OMT*, the codon-optimized *C. japonica CAS*, the codon-optimized *Berberis wilsonae STOX* on a yeast artificial chromosome vector	(R,S)-norlaudanosoline	Shake-flask fermentation	1.8 mg/L	Galanie and Smolke, 2015
(S)-Berberine	*S. cerevisiae*	*his3*::P_TEF1_-*Ps6OMT*-T_CYC1_, *leu2*::P_TEF1_-*PsCNMT*-T_CYC1_, *ura3*::P_TEF1_-*Ps4′OMT*-T_CYC1_, *trp1*::P_TEF1_-*CPR*-T_CYC1_, *lys2*::P_TEF1_-*PsBBE*-T_CYC1_.Expressing *9OMT* from *T. flavum* on a 2 μ plasmid. Expressing *P. somniferum BBE*, the codon-optimized *P. somniferum S9OMT*, the codon-optimized *C. japonica CAS*, the codon-optimized *B. wilsonae STOX* on a yeast artificial chromosome vector	(R,S)-norlaudanosoline	Shake-flask fermentation	6.5 μg/L	Galanie and Smolke, 2015
Opioid (Oxycodone, hydrocodone, etc)	*S. cerevisiae*	*ura3Δ*::P_GPD_-*T6ODM*-T_CYC1_, *his3Δ*::P_GPD_-*T6ODM*-T_CYC1_.Expressing the codon-optimized *P. somniferum T6ODM* and the codon-optimized *morB* from *P. putida* M10 on a yeast artificial chromosome vector	Thebaine	Parallel 0.25-L closed-batch fermentation	131 mg/L	Thodey et al., 2014
Opioid (14-hydroxycodeine, neopine, etc)		*ura3Δ*::P_GPD_-*T6ODM*-T_CYC1_, *his3Δ*::P_GPD_-*CODM*-T_CYC1_, *leu2Δ*::P_GPD_-*CODM*-T_CYC1_.Expressing the codon-optimized *P. somniferum T6ODM, P. somniferum CODM, P. putida* M10 *morA* and *morB^*E*160*G*^* on a yeast artificial chromosome vector	Thebaine	Parallel 0.25-L closed-batch fermentation	74 mg/L	Thodey et al., 2014
Opioid (14-hydroxycodeine, codeine, morphine, etc)	*S. cerevisiae*	*ura3Δ*::P_GPD_-*T6ODM*-T_CYC1_, *his3Δ*::P_GPD_-*CODM*-T_CYC1_, *leu2Δ*::P_GPD_-*CODM*-T_CYC1_.Expressing the codon-optimized *P. somniferum T6ODM, COR1.3-ER1*, and *CODM* on a yeast artificial chromosome vector	Thebaine	Parallel 0.25-L closed-batch fermentation	42 mg/L	Thodey et al., 2014
Thebaine	*S. cerevisiae*	Integration of the *ARO4^*Q*166*K*^, ARO7^*T*226*I*^, ARO10* and *TKL1* from *S. cerevisiae*, the four tetrahydrobiopterin (BH_4_) biosynthesis genes and recycling enzyme gene from *R. norvegicus*, the BH_4_ salvage enzyme dihydrofolate reductase (DHFR) gene and *tyrH^*R*37*E*/*R*38*E*/*W*166*Y*^* gene from *R. norvegicus*, the *dodc* from *P. putida*, the *NCS* from *C. japonica*, the *6OMT, CNMT, 4'OMT* and *CPR* from *P. somniferum, NMCH* from *E. californica*, the *DRS-DRR* from *P. bracteatum*, the chimeric protein gene of *E. californica CFS^1−83^* and *P. bracteatum* salutaridine synthase *SalSyn^92−504^*, salutaridine reductase gene from *P. bracteatum* and salutaridinol acetyltransferase gene from *P. somniferum*. Integration of additional copies of *R. norvegicus tyrH^*R*37*E*/*R*38*E*/*W*166*Y*^, P. somniferum 4'OMT* and *C. japonica NCS*	Dextrose	0.5-mL 96-well plate fermentation	6.4 μg/L	Galanie et al., 2015
Hydrocodone	*S. cerevisiae*	Integration of the *ARO4^*Q*166*K*^, ARO7^*T*226*I*^, ARO10*, and *TKL1* from *S. cerevisiae*, the four tetrahydrobiopterin (BH_4_) biosynthesis genes and recycling enzyme gene from *R. norvegicus*, the BH_4_ salvage enzyme dihydrofolate reductase (DHFR) gene and *tyr^*R*37*E*/*R*38*E*/*W*166*Y*^* from *R. norvegicus*, the *dodc* from *P. putida*, the *NCS* from *C. japonica*, the *6OMT, CNMT, 4'OMT* and *CPR* from *P. somniferum, NMCH* from *E. californica*, the *DRS-DRR* from *P. bracteatum*, the chimeric protein of	Dextrose	0.5-mL 96-well plate fermentation	0.3 μg/L	Galanie et al., 2015
		*E. californica* CFS^1−83^ and *P. bracteatum* salutaridine synthase SalSyn^92−504^, salutaridine reductase gene from *P. bracteatum* and salutaridinol acetyltransferase gene from *P. somniferum*. Integration of additional copies of *R. norvegicus tyrH^*R*37*E*/*R*38*E*/*W*166*Y*^, P. somniferum 4'OMT* and *C. japonica NCS*. Expression of *T6ODM* from *P. somniferum* and *morB* from *P. putida* M10 on a yeast artificial chromosome vector				
Thebaine	*E. coli-E. coli*	The dopamine producer: Δ*tyrR*. Expressing *tyrA^*fbr*^, tktA* and *ppsA* on a pCOLADuet-1 plasmid, expressing tyrosinase gene from *R. solanacearum* and *dodc* from *P. putida* on a pET32a plasmid. The (R,S)-THP producer: Expressing *mao* from *M. luteus* on a pGS21 plasmid.The (R,S)-reticuline producer: Expressing *CNMT* and *4'OMT* from *C. japonica* on a pET23a plasmid.The thebaine producer: Expressing the N-terminal truncated *SalS* from *P. somniferum* on a pET23a plasmid. Expressing *ATR2* from A*. thaliana, SalAT* and *SalR* from *P. somniferum* on a pCDF23 plasmid	Glycerol	Stepwise culture of four engineered strains	2.1 mg/L	Nakagawa et al., 2016
Hydrocodone	*E. coli-E. coli*	The dopamine, (R,S)-THP and (R,S)-reticuline producers are the same as that for the thebaine production. The thebaine producer: Expressing the N-terminal truncated *SalS* from *P. somniferum* on a pET23a plasmid.Expressing *ATR2* from A*. thaliana, SalAT* and *SalR* from *P. somniferum* on a pCDF23 plasmid. Expressing *T6ODM* from *P. somniferum* and *morB* from *P. putida* on a pCOLA2 plasmid.	Glycerol	Stepwise culture of four engineered strains	0.36 mg/L	Nakagawa et al., 2016

Resveratrol is sensitive to light and oxygen, which limits its bioavailability and bioactivity. Both properties can be enhanced by the substitution of hydroxyl groups with methoxy groups. Resveratrol methyltransferase from *A. thaliana* (Heo et al., 2017), *O. sativa* (Katsuyama et al., 2007a), *Sorghum bicolor* (Jeong et al., 2014; Li M. et al., 2016), *Vitis riparia* (Jeong et al., 2014), and *V. vinifera* (Wang Y. C. et al., 2015; Li M. et al., 2016; Kallscheuer et al., 2017; Wang et al., 2018) were used to convert resveratrol to **pinostilbene/pterostilbene** (Figure 5). The methyltransferase from *O. sativa* methylated the hydroxy groups at positions 3 and 5 of resveratrol to form pinostilbene/pterostilbene (Katsuyama et al., 2007a). The methylation of resveratrol by the methyltransferase from *S. bicolor* yielded pinostilbene as the major product and a very small amount of pterostilbene, whereas the methyltransferase from *V. riparia* showed very low methylation activity when resveratrol was used as a substrate (Jeong et al., 2014). The expression of the methyltransferase enzyme from *V. vinifera* or *A. thaliana* led to the production of the major product pterostilbene from resveratrol, and a very small amount of pinostilbene (Wang Y. C. et al., 2015; Li M. et al., 2016; Heo et al., 2017; Wang et al., 2018). The titer of pterostilbene (50 mg/L) produced from *p*-coumaric acid by the methyltransferase from *V. vinifera* (Wang Y. C. et al., 2015) was higher than that (10 mg/L) produced by the methyltransferase from *A. thaliana* (Heo et al., 2017), indicating that the methyltransferase from *V. vinifera* is more beneficial for the production of pterostilbene. The *de novo* biosynthesis of pinostilbene or pterostilbene from glucose was achieved for the first time by the introduction of the methyltransferase from *S. bicolor* or *V. vinifera*, respectively, into resveratrol producing yeast (Li M. et al., 2016). The engineered *S. cerevisiae* containing the methyltransferase from *S. bicolor* produced 5.52 mg/L of pinostilbene from glucose. The engineered *S. cerevisiae* containing the methyltransferase from *V. vinifera* produced 34.93 mg/L of pterostilbene and 1.96 mg/L of pinostilbene from glucose. Heo et al. (2017) also engineered *E. coli* for the *de novo* biosynthesis of pterostilbene from glucose by the overexpression of caffeic acid O-methyltransferase from *A. thaliana*, tyrosine ammonia lyase from *S. espanaensis, p*-coumarate: CoA ligase from *N. tabacum*, and stilbene synthase from *V. vinifera* in the L-Tyr-overproducing *E. coli* strain. The resulting *E. coli* produced 33.6 mg/L of pterostilbene from glucose in shake flask cultures containing additional L-methionine medium.

**Pinosylvin** is a natural resveratrol analog that lacks a hydroxyl group at the C-4 position. It is synthesized from L-Phe in three enzymatic steps (Figure 5). L-Phe is first transformed to cinnamic acid by L-Phe ammonia lyase. Subsequently, the enzyme 4-coumarate/cinnamate:coenzyme A ligase converts the cinnamic acid to cinnamoyl-CoA. Finally, the enzyme stilbene synthase catalyzes the stepwise condensation of three molecules of malonyl-CoA with one molecule of cinnamoyl-CoA, yielding one molecule of pinosylvin. The production of pinosylvin was achieved from L-Phe for the first time by the co-expression of the phenylalanine ammonia lyase gene from *Rhodotorula rubra*, the 4-coumarate:CoA ligase gene from *Lithospermum erythrorhizon*, the stilbene synthase gene from *A. hypogaea*, and the acetyl-CoA carboxylase gene from *C. glutamicum* (Katsuyama et al., 2007a). The resulting *E. coli* strain produced 20 mg/L pinosylvin from L-Phe. *De novo* biosynthesis of pinosylvin was achieved for the first time by the co-expression of the stilbene synthase gene from *Pinus strobus*, the 4-coumarate: CoA ligase gene from *S. coelicolor*, and the phenylalanine ammonia lyase gene from *P. crispum* (van Summeren-Wesenhagen and Marienhagen, 2015). The addition of cerulenin to increase the intracellular malonyl-CoA pools and the directed evolution of stilbene synthase from *P. strobus* further enhanced pinosylvin production. The final engineered *E. coli* strain produced 91 mg/L of pinosylvin from glucose after the addition of cerulenin and L-Phe (van Summeren-Wesenhagen and Marienhagen, 2015). Cerulenin is expensive and its use is not cost-effective for a large-scale fermentation process. In seeking a better alternative, the CRISPRi system was used to repress the *fadD* gene, which increased the availability of malonyl-CoA and resulted in the production of 47.5 mg/L of pinosylvin from glycerol (Liang et al., 2016). A rational modular design approach was used to improve pinosylvin production from glucose in *E. coli* (Wu et al., 2017a). The overall pinosylvin synthetic pathway was divided into two modules that were separated at cinnamic acid. The combined optimization of the transcriptional and translational levels of these two modules by modifying the plasmid copy numbers, promoter strength, and 5' region of the mRNA secondary structure increased the titer of pinosylvin by 16 times. The CRISPRi system was also used to repress *fabB, fabF, adhE, eno, fumC*, and *sucC* to improve pinosylvin production. The resulting *E. coli* produced 281 mg/L of pinosylvin, which was the highest titer directly obtained from glucose without the provision of supplementation with any additional precursors (Wu et al., 2017a). The metabolic engineering strategy based on the post-translational modification of proteins was used to optimize the lysine acylation of the stilbene synthase and to improve pinosylvin production by 220% (Xu et al., 2018).

**Piceatannol** is another natural resveratrol analog with an additional phenolic hydroxyl group at the C-3 position. It can be synthesized from caffeic acid or resveratrol (Figure 5). *E. coli* W endogenous non-P450 monooxygenase (HpaBC) catalyzed the *ortho*-hydroxylation of resveratrol to piceatannol, which resulted in the maximal titer of 1.2 g/L from resveratrol with a nearly 100% molar yield (Lin and Yan, 2014). An engineered *C. glutamicum* was constructed for the production of pinosylvin or piceatannol from cinnamic acid or caffeic acid, respectively, by the introduction of stilbene synthase from *A. hypogaea* (Kallscheuer et al., 2016).

## Production of Benzylisoquinoline Alkaloids

Benzylisoquinoline alkaloids (BIAs) are a large family of L-tyrosine-derived plant-specialized compounds with a variety of therapeutic uses. This class of compounds includes the opioid analgesics morphine and codeine, the antibiotics sanguinarine and berberine, the muscle relaxants (+)-tubocurarine and papaverine, and the cough suppressant noscapine. BIA synthesis begins with the condensation of dopamine and 4-hydroxyphenylacetaldehyde or 3,4-di hydroxyphenylacetaldehyde to form (S)-norcoclaurine or (S)-norlaudanosoline ((S)-NorLaud, also known as (S)-tetrahydropapaveroline, (S)-THP) in engineered microorganisms (Figure 5, Table 4) (Diamond and Desgagne-Penix, 2016; Narcross et al., 2016b). Both (S)-norcoclaurine and (S)-norlaudanosoline are unstable end-products, because they are subject to enzymatic oxidation as well as spontaneous oxidation at alkaline pHs. Thus, reticuline is frequently used as the key branch-point intermediate for the *de novo* synthesis of BIAs. **Reticuline** was produced in *E. coli* for the first time by the overexpression of monoamine oxidase (Mao) from *M. luteus*, norcoclaurine synthase (NCS) from *Coptis japonica*, norcoclaurine 6-O-methyltransferase (6OMT) from *C. japonica*, coclaurine-N-methyltransferase (CNMT) from *C. japonica*, and 3'-hydroxy-N-methylcoclaurine-4'-O-methyltransferase (4'OMT) from *C. japonica* (Figure 5) (Minami et al., 2008). Since some plant enzymes are not well-expressed in *E. coli* in an active form, various types of BIAs were then synthesized from reticuline using engineered *S. cerevisiae*. Co-cultures of *E. coli* expressing the reticuline biosynthetic genes and *S. cerevisiae* expressing *C. japonica* CYP80G2 and CNMT, or the *C. japonica* berberine bridge enzyme (BBE) were used to produce **magnoflorine** or **scoulerine**, respectively, from dopamine (Figure 5) (Minami et al., 2008). The overall yield of magnoflorine or scoulerine from dopamine was 1.9 or 2.2%, respectively. After optimizing the fermentation conditions and the gene copy numbers, the reticuline titer generated from dopamine was increased by 5-fold to 54 mg/L (Kim J. S. et al., 2013). Recently, this group comparatively analyzed the effect of the three methyltransferases on the biosynthesis of reticuline and found that the combination of 6OMT from *P. somniferum*, CNMT from *P. somniferum*, and 4'OMT from *C. japonica* was the most suitable combination for reticuline production (Matsumura et al., 2017). The *de novo* biosynthesis of reticuline was achieved for the first time by the co-expression of tyrosinase from *Ralstonia solanacearum*, DOPA decarboxylase (Dodc) from *P. putida* strain KT2440, Mao from *M. luteus*, NCS from *C. japonica*, 6OMT from *C. japonica*, CNMT from *C. japonica*, and the 4'OMT from *C. japonica* in the L-Tyr overproducing *E. coli* strain (Nakagawa et al., 2011). The final strain produced 46 mg/L (S)-reticuline from glycerol in a fed-batch fermentation process. Then, this group further improved the (S)-reticuline titer to 163.5 Mg/L by an engineered *E. coli* strain EM353, which possessed a tyrosine-overproducing pathway, a pathway producing dopamine from L-Tyr along with the BH4-synthesis pathway, and a pathway producing (S)-reticuline from dopamine (Matsumura et al., 2018). In the above studies, tetrahydropapaveroline (THP), a reticuline precursor, is synthesized by Mao and Dodc. Recently, an insect *Bombyx mori* 3,4-dihydroxyphenylacetaldehyde synthase (DHPAAS) and its variants with Phe79Tyr, Tyr80Phe, and Asn192His were identified to replace Mao and Dodc for direct production (R,S)-THP in a single enzyme system from L-DOPA (Vavricka et al., 2019).

*S. cerevisiae* is a more attractive host for BIA synthesis, due to its superior ability to express the plant enzymes and the cytochrome P450s, which are prevalent in BIA biosynthesis. The synthesis of reticuline in *S. cerevisiae* was initially achieved with a yield of 10% from norlaudanosoline by the over-expressions of *P. somniferum* 6OMT, CNMT, and 4'OMT (Hawkins and Smolke, 2008). *S. cerevisiae* strains were also engineered for the production of the protoberberine intermediate (S)-canadine and the morphinan intermediate salutaridine from (R,S)-norlaudanosoline (Hawkins and Smolke, 2008). The yield of reticuline obtained from norlaudanosoline in *S. cerevisiae* was then increased to 20% (Fossati et al., 2014). These two research groups constructed a *de novo* biosynthetic pathway for producing reticuline from glucose *via* norcoclaurine and reported their results at the same time (DeLoache et al., 2015; Trenchard et al., 2015). To solve the issue of the poor activity of tyrosine hydroxylase in yeast, an enzyme-coupled biosensor of L-DOPA was developed. Using this sensor, a mutant (W13L/F309L) of CYP76AD1 from *Bete vilgaris* was obtained to improve its L-DOPA yield by 2.8-fold via directed evolution (DeLoache et al., 2015). The co-expression of the feedback-insensitive Aro4, the CYP76AD1 variant, DOPA decarboxylase (DODC) from *P. putida*, a newly identified NCS from *P. somniferum*, 6OMT, CNMT, and 4′OMT from *P. somniferum*, and the cytochrome P450 N-methylcoclaurine hydroxylase (NMCH) from *Eschscholzia californica* in *S. cerevisiae* resulted in the *de novo* production of 80.6 μg/L of reticuline from glucose (DeLoache et al., 2015). In the study by the research group led by Smolke, the mammalian tyrosine hydroxylase mutant (W166Y/R37E/R38E) from *Rattus norvegicus*, the four tetrahydrobiopterin (BH_4_) biosynthesis and recycling enzymes from *R. norvegicus*, and bacterial DOPA decarboxylase from *P. putida* were introduced in the L-Tyr overproducing *S. cerevisiae* strain for the production of norcoclaurine (Trenchard et al., 2015). The co-expression of optimized NCS from *C. japonica*, the three methyltransferases (6OMT, CNMT, and 4′OMT) from *P. somniferum*, the cytochrome P450 (CYP80B1) from *E. californica*, and its reductase partner (CPR) from *P. somniferum* in the aforementioned engineered *S. cerevisiae* strain resulted in the *de novo* production of 19.2 mg/L of reticuline from glucose in a shake flask fermentation process (Trenchard et al., 2015).

**Noscapine** has long been used as a cough suppressant. Due to its long-established safety record, noscapine is a compelling candidate anti-cancer drug and has been used off-label to treat cancers in some countries. Noscapine is synthesized from L-Tyr through scoulerine (Figure 5). The synthetic pathway of noscapine with the 10-gene cluster was discovered in *P. somniferum* (Winzer et al., 2012). A 14-step biosynthetic pathway for the production of noscapine from norlaudanosoline was engineered for the first time in yeast (Li and Smolke, 2016). After the optimization of the expression of 16 plant enzymes involved in this biosynthetic pathway, the engineered *S. cerevisiae* strain produced 1.64 mM of noscapine. This group subsequently reported the *de novo* production of noscapine in *S. cerevisiae*, through the reconstruction of a biosynthetic pathway comprising 37 enzymes from plants, bacteria, mammals, and yeasts (Li Y. et al., 2018). After engineering rate-limiting pathway enzymes, optimizing enzyme expression levels, introducing modifications to the endogenous yeast metabolism to enhance NADPH availability, and optimizing fermentation conditions, the noscapine titer was improved by over 18,000-fold to ~2.2 mg/L.

**Sanguinarine** is a BIA with recognized antimicrobial activities. It may have potential as an anti-neoplastic drug. Fossati et al. constructed a 10-gene plant pathway in *S. cerevisiae* that allowed the production of the sanguinarine precursor dihydrosanguinarine from (R,S)-norlaudanosoline, which resulted in a yield of 1.5% (Figure 5) (Fossati et al., 2014). The spontaneous oxidation of dihydrosanguinarine to sanguinarine was observed in this study. The 10 enzymatic steps included four reactions catalyzed by plant cytochrome P450s (Fossati et al., 2014). After optimizing the expression of the cytochrome P450s (including P450 enzymatic variants, the pairing of CPRs with P450 enzymatic variants, gene number copy and promoter) and culture conditions, 80 μg/L sanguinarine was produced from (R, S)-norlaudanosoline in the engineered *S. cerevisiae* (Trenchard and Smolke, 2015). This titer was achieved by the expression of *P. somniferum 6OMT, CNMT, 4'OMT, BBE, CPR, TNMT*, and *MSH, E. californica CFS, STS*, and *P6H*, and *A. thaliana ATR1*. Mining of transcriptome libraries identified the superior cytochrome P450 enzymes CFS and SPS (Narcross et al., 2016a). They improved the level of molar conversion of norlaudanosoline to dihydrosanguinarine to 10%. This study demonstrated that the mining of transcriptome libraries combined with gene synthesis is an effective strategy for pathway engineering.

Hawkins and Smolke reconstructed the biosynthetic pathway for the berberine precursor **(S)-canadine** in *S. cerevisiae* to achieve the microbial biosynthesis of (S)-canadine from (R,S)-norlaudanosoline (Figure 5) (Hawkins and Smolke, 2008). Then, this group optimized cytochrome P450 oxidoreductase (canadine synthase CAS), scoulerine 9-O-methyltransferase S9OMT, and culture conditions, which improved the canadine and berberine production from (R,S)-norlaudanosoline to 1.8 and 6.5 mg/L, respectively (Galanie and Smolke, 2015). Their studies also demonstrated that canadine can be easily and spontaneously converted to berberine. The *de novo* synthesis of berberine has not been achieved in microorganisms to date. The abovementioned strategies as reported by Li Y. et al. (2018), which were used for the *de novo* synthesis of noscapine, can be used for berberine production, because berberine and noscapine have the same precursor, i.e., (S)-canadine.

Thodey et al. (2014) engineered *S. cerevisiae* strains to convert thebaine to codeine, morphine, hydromorphone, hydrocodone, and oxycodone, by expressing the thebaine 6-O-demethylase (T6ODM), codeine-O-demethylase (CODM), and codeinone reductase (COR1.3) from *P. somniferum* and MorAB from *P. putida* M10. The authors improved the total **opioid** titers to 130 mg/L by titrating gene copy number, applying a spatial engineering strategy, and optimizing culture conditions. In another study, a seven-gene pathway was constructed in *S. cerevisiae* for the production of **codeine** from (R)-reticuline (Fossati et al., 2015). These seven genes included salutaridine synthase gene (*SAS*), salutaridine reductase gene (*SAR*), salutaridinol acetyltransferase gene (*SAT*), and *T6ODM, COR1.3*, and *CODM* from *P. somniferum*. Another research group engineered 21-gene and 23-gene pathways in *S. cerevisiae* for the *de novo* production of **thebaine** and **hydrocodone**, respectively, from dextrose (Galanie et al., 2015). The genes of the 21-gene pathway for thebaine synthesis composed 5 modules: (1) precursor overproduction modular, (2) tetrahydrobiopterin (BH_4_) modular, (3) (S)-norcoclaurine modular, (4) (S)-reticuline modular, (5) thebaine modular (Figure 5, Table 4). And they also demonstrated that the (S)-reticuline modular is the key pathway for opioid production (Galanie et al., 2015). Integrating additional gene copies of the (S)-reticuline modular increased reticuline production to 82 μg/L from 20 μg/L. The integration of 21 genes of the thebaine synthetic pathway and additional copies of the (S)-reticuline modular (TyrH, 4'OMT, and NCS) in *S. cerevisiae* resulted in the production of 6.4 μg/L thebaine. The authors also described that the overexpression of T6ODM from *P. somniferum* and MorB from *P. putida* M10 in a yeast artificial chromosome vector in the above thebaine producing yeast stain resulted in the production of ~0.3 μg/L of hydrocodone (Galanie et al., 2015). A stepwise culture strategy was used for the *de novo* production of opiates from glycerol using four *E. coli* strains (Nakagawa et al., 2016). These four *E. coli* strains included dopamine, (R,S)-THP, (R,S)-reticuline, and thebaine producing *E. coli* strains. The stepwise culture of the four engineered *E. coli* strains yielded 2.1 mg/L of thebaine from glycerol, which corresponded to a 300-fold increase from the yield observed with the developed yeast system (Nakagawa et al., 2016). After the introduction of *T6ODM* obtained from *P. somniferum* and *morB* from *P. putida* into the thebaine producing *E. coli* strain, 0.36 mg/L of hydrocodone was produced using the four-step culture system (Nakagawa et al., 2016). A neopinone isomerase gene *(NISO*) was recently identified from opium poppy (*P. somniferum*) (Dastmalchi et al., 2019b). Overexpression of this *NISO* in an engineered yeast significantly improved the production of codeine and hydrocodone, respectively. Recently, nine benzylisoquinoline alkaloid uptake permease genes (*BUPs*) were also identified from *P. somniferum* (Dastmalchi et al., 2019a). Overexpression of *BUP* in an engineered yeast further enhanced the production of (S)-reticuline, thebaine, codeine, and morphine, respectively.

## Concluding Remarks and Future Prospects

With the rapid development of metabolic engineering and synthetic biology, as well as the increased knowledge regarding the native biosynthetic pathways of aromatic natural compounds, heterologous biosynthesis of aromatic products in microorganisms has become a promising alternative for the production of aromatics and their derivatives. However, their titers and yields are still too low for industrial production. The following may be new directions that can enhance the production of aromatic amino acid derivatives.

First, many aromatic compounds have anti-microbial activity and are highly toxic to microbial host cells, thus limiting the microbial production of aromatic chemicals. Adaptive laboratory evolution (ALE) is a general approach for improving the tolerance of host cells. However, t improvements in tolerance cannot guarantee an increase in yield. The biosensor-driven ALE approaches can rapidly screen the highly efficient evolved strain from the ALE libraries tolerant to the aromatic compounds. Some biosensors of aromatic compounds have been developed (Li et al., 2017; Wang et al., 2017). Shikimic acid and resveratrol biosensors have been developed and applied to increase the production of shikimic acid (Li et al., 2017) and resveratrol (Xiong et al., 2017), respectively. Liu et al. (2017) developed an L-Phe biosensor and applied the biosensor to improve L-Phe production, by screening hyperproducing strains from a ribonucleotide binding site library and random mutagenesis library.

Second, whole-cell biocatalysis may be a powerful strategy for the production of aromatic chemicals. The whole-cell biocatalysis processes consist of two stages: cell culture and the conversion of the substrates (Lin and Tao, 2017). After cells are cultured, cells are harvested and then suspended in the desired buffer to convert substrates into products. Because the whole-cell biocatalysis process separates the cell growth from the formation of target products, it is unnecessary to consider the cytotoxicity of products. It has been successfully used for the production of L-PLA from L-Phe (Hou et al., 2019), D-phenylglycine from L-Phe (Zhou Y. et al., 2017), rosmarinic acid from caffeic acid (Jiang et al., 2016), L-DOPA from hydroxytyrosol (Li et al., 2019b), and caffeic acid from *p*-coumaric acid (Furuya and Kino, 2014). It can also be used for the *de novo* biosynthesis of products when cells harbor the *de novo* biosynthetic pathway. Using whole-cell biocatalysis of the engineered *C. glutamicum* resulted in the highest shikimic acid titer and yield attained by microbial production reported so far (141 g/L and 0.49 g of shikimic acid per g of glucose (Kogure et al., 2016).

Third, because regulation mechanisms of aromatic amino acid formation have been quite well-known and thus do no longer present obstacles in increasing the aromatic amino acid availability. The identification of better enzymes of the heterogeneous pathway and the further improvement of these enzymes will play important roles in further improving the production of aromatic compounds. A mutant of *A. thaliana* 4CL1 exhibiting a higher catalytic efficiency was obtained by directed evolution via error-prone PCR (Xiong et al., 2017). The use of this mutant improved resveratrol production by 4.7-fold in *E. coli*.

Fourth, modular co-cultures might be a useful approach for improving the production titer of aromatic compounds. This approach divides a complete biosynthetic pathway into separate serial modules and uses selected microbial strains to accommodate individual modules for biosynthesis. There are a number of main benefits of using this approach. These include: (1) reduction of the metabolic burden on each host strain; (2) allowing the selection of a suitable host strain and culture conditions for each module that meets the requirements of specific biosynthetic steps; (3) reduction in the undesirable interference from different pathways; (4) maintenance of an easy balance of the biosynthetic pathway among individual pathway modules, by simply changing the strain-to-strain ratio; (5) high-efficiency utilization of complex materials containing multiple active substrates; and (6) support provided for the plug-and-play biosynthesis of various target products (Zhang and Wang, 2016). Co-culture engineering strategies can significantly improve the production of salidroside (Liu X. et al., 2018) and resveratrol (Camacho-Zaragoza et al., 2016). BIAs are tyrosine derivatives. Strategies for improving the production of tyrosine in *E. coli* and *S. cerevisiae* are similar, but the highest published titers of aromatic amino acids in *E. coli* are currently an order of magnitude higher than that in *S. cerevisiae*. Moreover, plant enzymes and cytochrome P450s are more efficiently expressed in *S. cerevisiae* than in *E. coli*. Thus, the modular co-culture of *E. coli* and *S. cerevisiae*—during which *E. coli* expresses the microbial genes for synthesizing (S)-THP and *S. cerevisiae* express the plant enzymes and cytochrome P450s required for synthesizing BIAs—might be an efficient strategy for the biotechnological production of BIAs. Minami et al. (2008) constructed a co-culture system involving *E. coli* and *S. cerevisiae* for the production of magnoflorine or scoulerine from dopamine.

Fifth, most of the studies that have been discussed used the plasmid system to achieve the expression of heterologous pathways. Drawbacks that include the instability and the necessity of use of antibiotics in plasmid expression systems limit the industrial use of plasmid expression systems. To overcome these drawbacks associated with plasmid expression systems, a chemically inducible chromosomal evolution (CIChE) system (Tyo et al., 2009) and an auxotrophic system (Shukal et al., 2019) were developed to achieve high gene copy expression. However, the constructed CIChE strains contained an antibiotic resistance marker (chloramphenicol resistance) (Tyo et al., 2009). Subsequently, we modified CIChE strains and developed an approach that involves triclosan inducible chromosomal evolution (Chen et al., 2013). We applied this approach to engineer a lycopene (Chen et al., 2013) and shikimic acid (Cui et al., 2014) hyperproducing *E. coli* strains that do not carry a plasmid or antibiotic marker.

The final, as in the cases for developing strains overproducing other compounds, systems metabolic engineering will also be used for developing microbial strains efficiently producing various aromatic chemicals. This approach, which integrates metabolic engineering with systems biology, might represent another efficient strategy to improve the production of aromatic compounds. Comparative analyses using omics might lead to the identification of unknown bottlenecks and help overcome them, and might enable the use of reverse engineering, i.e., the application of novel targets in existing aromatic-producing strains. Although systems metabolic engineering has successfully been applied to engineer microorganisms as a way of improving the production of chemicals (Cho et al., 2015; Shen et al., 2016; Li and Liu, 2017), there are few reports regarding the production of aromatic compounds.

## Author Contributions

Y-PS, F-XN, and Y-BH provided draft text for the section Production of Aromatic Amino Acid Derivatives. Z-BY provided draft text for the section Production of Stilbens. LF provided draft text for the section Production of Benzylisoquinoline Alkaloids. J-ZL conceived, reviewed the literature and extracted data, plotted figures and wrote the manuscript. All authors read and approved the final manuscript.

## Conflict of Interest

The authors declare that the research was conducted in the absence of any commercial or financial relationships that could be construed as a potential conflict of interest.
